# Molecular Basis of Simalikalactone D Sensitivity in Triple-Negative Breast Cancer Cells

**DOI:** 10.3390/biom15111561

**Published:** 2025-11-06

**Authors:** Annelis O. Sánchez-Álvarez, Joshua Nieves-Reyes, Gabriel Borges-Vélez, Josué Pérez-Santiago, Misael Rivera-García, Stella Alicea-Ayala, Claudia Ospina-Millan, Fatima Valiyeva, Pablo E. Vivas-Mejia

**Affiliations:** 1 Comprehensive Cancer Center, University of Puerto Rico, San Juan 00936, Puerto Rico or ansanchez@cccupr.org (A.O.S.-Á.);; 2 Department of Biochemistry, University of Puerto Rico, Medical Sciences Campus, San Juan 00935, Puerto Rico; joshua.nieves7@upr.edu; 3 Department of Biology, University of Puerto Rico, Rio Piedras Campus, San Juan 00927, Puerto Rico; 4 Department of Natural Sciences and Mathematics, Inter American University of Puerto Rico, Bayamon Campus, Bayamón 00957, Puerto Rico

**Keywords:** triple negative breast cancer, apoptosis, migration, EGFR, JAK/STAT, ITGB1, Simalikalactone D

## Abstract

**Background/Objective:** Triple-negative breast cancer (TNBC) is an aggressive subtype of breast cancer (BC) lacking targeted therapies and characterized by high tumor heterogeneity. In this study, we evaluated the anticancer activity and mechanistic profile of Simalikalactone D (SKD), a quassinoid compound derived from the endemic Puerto Rican tree *Simarouba tulae*, in three TNBC cell lines, MDA-MB-468, MDA-MB-231, and SUM-149. **Methods:** MDA-MB-468, MDA-MB-231 and SUM-149 TNBC cells were evaluated for cell viability, proliferation and migration following SKD treatment. Phospho-antibody array, proteomics, and Western blot analyses were used to explore the SKD mechanism of action in MDA-MB-468 and MDA-MB-231 cell lines. Molecular docking was performed to assess SKD’s interaction with potential intracellular targets. **Results:** SKD exerted a concentration-dependent effect on the three cell lines. However, MDA-MB-468 cells exhibited an IC_50_ of 67 nM, while the IC_50_ values for MDA-MB-231 and SUM-149 were 422 nM and 598 nM, respectively. In MDA-MB-468 cells, 100 nM of SKD induced apoptosis, evidenced by the activated caspase-3 activity, PARP-1 cleavage and decrease in Bcl-2 and survivin protein levels. Sublethal SKD (25 nM) impaired migration in MDA-MB-231 cells and reduced proliferation and motility in SUM149 cells. A 6 h SKD treatment markedly reduced phosphorylation of apoptosis-related proteins (p53, BAD, DAXX, AKT1, JUN) and Jak/STAT pathway components, indicating early disruption of intracellular signaling prior to phenotypic changes. Proteomic profiling showed distinct pathway alterations in both MDA-MB-468 and MDA-MB-231 cells, with reduced Integrin β1 (ITGB1) levels emerging as a shared effector. This suggests that SKD broadly disrupts cell adhesion and migration independently of apoptosis-driven cell death. Western blot validation confirmed reduced ITGB1 protein levels across all three TNBC cell lines examined. In silico docking confirmed favorable binding affinities of SKD to both EGFR (ΔG = −6.718 kcal/mol) and STAT4 (ΔG = −8.481 kcal/mol). **Conclusions:** Overall, our findings suggest that SKD is a potent anticancer agent in a subgroup of TNBC cells.

## 1. Introduction

Triple-negative breast cancer (TNBC) is an aggressive breast cancer subtype, accounting for 15–20% of all cases [1]. Unlike other subtypes, TNBC lacks the expression of receptors for estrogen (ER), progesterone (PR), and human epidermal growth factor (HER2), making it unresponsive to targeted hormonal therapies and leading to limited treatment options, leaving chemotherapy as the primary treatment option—often with limited success and significant toxicity [2]. TNBC disproportionately affects women of African ancestry, including African American and Afro-Caribbean populations, and is associated with a higher likelihood of recurrence, metastasis, and poor prognosis [3,4]. Growing interest in plant-derived compounds has driven the search for new therapeutics with selective anticancer activity and novel mechanisms of action. One such compound is Simalikalactone D (SKD) (Supplementary Figure S1A), a quassinoid isolated from Simarouba tulae, an endemic tree of Puerto Rico. Although SKD has demonstrated anticancer properties in other models (ovarian and breast), its mechanism of action in TNBC remains largely unknown [5].

In this study, we investigated the biological and molecular effects of SKD in three genotypically distinct TNBC cell lines: MDA-MB-468, derived from an African American woman with high EGFR expression and a mutant (gain-of-function) TP53 (R273H); MDA-MB-231, derived from a Caucasian woman with moderate EGFR levels and a different TP53 GOF mutation (R280K); and SUM-149, derived from an inflammatory breast cancer (IBC) tumor with constitutive activation of the EGFR pathway and a TP53 GOF mutation (Met237Ile) [6,7,8,9,10,11,12]. Key molecular differences between these models, MDA-MB-231, MDA-MB-468 and SUM-149, underscore significant molecular heterogeneity inherent to TNBC evident in the distinct subtypes with different oncogenic drivers and phenotypes. MDA-MB-231 cells are claudin-low, mesenchymal-like, and carry an oncogenic KRAS G13D mutation along with a BRAF mutation, supporting their dependency on the RAS/MAPK pathway and contributing to their spindle-shaped morphology and aggressive invasiveness [13,14,15,16,17,18]. In contrast, MDA-MB-468 cells display a basal-like, epithelial-like phenotype and harbor a PTEN mutation, resulting in constitutive activation of the PI3K/AKT pathway. Like MDA-MB-468, SUM149 is classified as basal A, expressing genes such as CK5/6, CK14, and EGFR, and also harboring a BRCA1 mutation [12]. These genotypic and phenotypic differences, KRAS/BRAF versus PTEN and/or BRCA1 mutations, mesenchymal versus epithelial/hybrid morphology, and varying pathway dependencies, make these cell lines excellent models for evaluating how diverse TNBC subtypes respond to targeted therapies such as SKD [13,17,19]. These intrinsic differences affect not only cellular behavior but also drug sensitivity and pathway dependency, making them valuable models for dissecting genotype-specific therapeutic responses in TNBC. These cell lines represent divergent TNBC phenotypes and allow for the comparison of SKD’s biological effects across molecular backgrounds.

## 2. Materials and Methods

### 2.1. Isolation and Identification of SKD and General Experimental Procedures

The method for the purification of SKD has already been described and was followed as reported with some modifications [5]. The chloroform extract will be chromatographed on Si gel with 5% methanol in chloroform to obtain 23 fractions that will be analyzed by thin-layer chromatography (TLC) and nuclear magnetic resonance (NMR). Fractions 8 to 11 were combined and purified by column chromatography with a mixture of methanol in chloroform to obtain 13 subfractions. Subfractions containing impure SKD were purified by flash reverse-phase chromatography using water/methanol solvent gradients from 50% water to 0% water in methanol (Supplementary Figure S1B) to obtain 210 mg of white solid. White solid was identified as SKD by NMR. All solvents and reagents were purchased from Sigma Aldrich. Fractions were concentrated using a Buchi Rotavapor R-300. Column chromatography was performed using silica gel (35–75 mesh and 200–425 mesh). TLC analyses were carried out using Analtech glass precoated Si gel plates (250 μm thick, 60 Å pore diameter) with a UV indicator. Spots were detected on TLC under UV light using a UV fluorescent Spectroline E Series Ultraviolet lamp (254 nm), followed by staining with iodine. Flash chromatography was performed on a Biotage^®^ Selekt system equipped with a Biotage^®^ Sfar pre-packed C18 D Duo column (100 Å, 30 μm, 12 g). NMR data were recorded on a Bruker Ascend 500 MHz spectrometer (Bruker TopSpin version 4.5.0, Bruker Inc., Billerica, MA, USA) operating at 500 MHz for ^1^H-NMR and 125 MHz for ^13^C-NMR. All ^1^H-NMR and ^13^C-NMR chemical shifts are referenced to residual CHCl_3_ in the deuterated solvent (7.26 ppm for 1H-NMR and 77.0 ppm for ^13^C-NMR). The NMR data analysis was performed using JEOL JASON software version 4.0.

### 2.2. Cell Lines and Cell Culture Maintenance

The human triple-negative breast cancer cell lines MDA-MB-231, MDA-MB-468, and SUM-149 were purchased from the American Type of Culture Collection (ATCC, Manassas, VA, USA). MDA-MB-231 cells were cultured in RPMI 1640 medium (Sigma-Aldrich, St. Louis, MO, USA) supplemented with 10% fetal bovine serum (FBS), (Thermo Fisher, Waltham, MA, USA), 1% penicillin–streptomycin cocktail (Thermo Fisher, Waltham, MA, USA), and 5 mg of insulin (Sigma-Aldrich, St. Louis, MO, USA) in 500 mL of media. The complete medium was filtered through a 0.22 μm filter to ensure sterility. MDA-MB-468 cells were cultured in high glucose Dulbecco’s Modified Eagle’s Medium (DMEM HG) medium (Sigma-Aldrich, St. Louis, MO, USA) supplemented with 10% FBS (Thermo Fisher, Waltham, MA, USA), 1% penicillin-streptomycin cocktail (Thermo Fisher, Waltham, MA, USA), 1% sodium pyruvate (Sigma-Aldrich, St. Louis, MO, USA), and 1% HEPES buffer (Sigma-Aldrich, St. Louis, MO, USA) in 500 mL of media. SUM-149 cells were cultured in Ham’s F12, 1.0 mM stable Glutamine, 1.0 mM Sodium pyruvate, 1.1 g/L NaHCO_3_ (Cytion, Eppelheim, Deutschland) supplemented with 5 μg/mL insulin, 2 μg/mL hydrocortisone, 10% FBS (Thermo Fisher, Waltham, MA, USA), 1% penicillin-streptomycin cocktail (Thermo Fisher, Waltham, MA, USA), in 500 mL of media. Cultures were maintained in a humidified incubator at 37 °C with 5% CO_2_ under standard culture conditions.

### 2.3. Cell Viability Assay

Cell viability was assessed using the Alamar Blue assay. MDA-MB-231, MDA-MB-468, and SUM-149 cells were seeded in 96-well plates at a density of 3500 cells per well in 100 µL of complete medium. After 24 h of initial seeding, cells were treated with Simalikalactone D (SKD) at varying concentrations and incubated for 72 h. After 72 h of incubation, a 1:10 (*v*/*v*) ratio of AlamarBlue reagent (Thermo Fisher Scientific, Waltham, MA, USA) to culture medium was prepared, and 100 µL of the mixture was added to each well of the 96-well plate. The plates were then incubated at 37 °C for 4 h to allow for color development. Absorbance was measured at 570 nm with a reference wavelength of 600 nm using a plate reader. The relative viability of treated cells was calculated as a percentage of control cells.

### 2.4. Cell Growth and Proliferation Studies

To assess the effect of SKD on cell proliferation, MDA-MB-231, MDA-MB-468 and SUM-149 cells were seeded at a density of 4.5 × 10^4^ cells/mL in clear 6-well plates (Eppendorf, Hamburg, Germany) and allowed to adhere overnight under standard cell culture conditions (37 °C, 5% CO_2_). The next day, cells were treated with SKD at final concentrations of 25 nM and 100 nM for 24, 48, and 72 h. At each point, cell proliferation was assessed by detaching cells from the wells using trypsin-EDTA solution (Sigma-Aldrich, St. Louis, MO, USA), followed by neutralization with complete medium. The cells were then collected and counted using a hemocytometer to determine the cell concentration (cells/mL) for each treatment group.

### 2.5. Wound Healing Assay for Cell Migration

Cell migration was assessed using a wound healing assay [20]. MDA-MB-231, MDA-MB-468 and SUM-149 cells were seeded at a density of 7.5 × 10^4^ cells/mL in 6-well plates and allowed to adhere overnight. The following day, cells were treated with 25 nM of SKD. After the treatment period, a sterile 200 μL pipette tip was used to create a straight scratch down the center of each well to simulate a “wound.” The wells were then gently rinsed with Dulbecco’s Phosphate-Buffered Saline (PBS) to remove any detached cells and debris. Following scratch creation, the wells were supplemented with fresh medium containing the appropriate treatment conditions. Wound closure was monitored by capturing images at 0, 24, 48 and 72 h post-scratch using a Nikon Eclipse TS100 microscope. The extent of cell migration into the wound area was quantified by measuring the wound width at each time point using Image J software v1. 54g. The percentage of wound closure was calculated using the following formula:

$$\mathrm{Wound}\;\mathrm{Closure}\;\mathrm{Ratio}\;=\;1-(\frac{Mean\;of\;initial\;wound\;width-Mean\;of\;current\;wound\;width}{Mean\;of\;initial\;wound\;width})$$

### 2.6. Caspase-3 Activity Assay

Apoptosis was assessed by measuring caspase-3 activity using the fluorometric Caspase-3 Assay Kit (cat# ab39383, Abcam, Waltham, MA, USA) according to the manufacturer’s instructions. Briefly, protein lysates were prepared from treated (100 nM SKD for 48 h) and untreated (PBS) cells, and protein concentration was determined using a BCA assay. A total of 100 μg of protein lysate was added to each well of a black 96-well plate to minimize background fluorescence. Each sample was then mixed with 50 μL of Reagent Buffer containing 10 mM DTT (dithiothreitol) to stabilize the enzyme activity. Subsequently, 5 μL of the caspase-3-specific fluorogenic substrate DEVD-AFC (50 μM; AFC: 7-amino-4-trifluoromethyl coumarin) was added to each well. The plates were incubated for 2 h at 37 °C in the dark to allow substrate cleavage by active caspase-3. Fluorescence was measured at an excitation wavelength of 400 nm and an emission wavelength of 505 nm using a Varioskan LUX multimode microplate reader (Thermo Fisher Scientific, Waltham, MA, USA). Caspase-3 activity was quantified based on the fluorescence intensity relative to untreated controls.

### 2.7. Western Blot Analysis

MDA-MB-231, MDA-MB-468 and SUM-149 cells were seeded at a density of 4.5 × 10^4^ cells/mL in 10 cm Petri dishes and allowed to attach overnight. The next day, cells were treated with 100 nM SKD for 48 h. Following treatment, cells were harvested and lysed by incubation on ice for 30 min in a cold lysis buffer. During this incubation, lysates were periodically vortexed to ensure thorough mixing. The supernatant was collected after centrifugation at 13,000 rpm for 20 min at 4 °C, and protein concentration was determined using the BCA assay (Bio-Rad Laboratories, Hercules, CA, USA), following the manufacturer’s protocol. For each sample, 50 µg of protein was mixed with lysis buffer and loading dye, then resolved by SDS-PAGE on a 10–12% gel according to protein’s molecular weight. Proteins were then transferred to PVDF membranes (MilliporeSigma, St. Louis, MO, USA) at 100 V for 1 h. Membranes were blocked with 5% non-fat milk in TBST (Tris-buffered saline with 0.1% Tween-20) for 1 h at room temperature. Primary antibodies against PARP (cat#9542S), c-Caspase-3 (cat#9661), BCL-2 (cat#2872), p53 (cat#2527), survivin (cat#2803), Caspase 3 (cat#9665), B-actin (cat#3700) (Cell Signaling Technology, Danvers, MA, USA), and ITGB1 (cat#MAB1778) (R&D Systems, Minneapolis, MN, USA), were applied overnight at 4 °C. After three washes with TBST, membranes were incubated with the corresponding HRP-conjugated secondary antibodies (1:5000) for 1 h at room temperature. For signal detection, enhanced chemiluminescence (ECL) reagents (e.g., SuperSignal West Pico Plus, Thermo Fisher Scientific, Waltham, MA, USA) were applied to the membranes, and the blots were visualized using a Chemidoc imaging system (Bio-Rad Laboratories, Hercules, CA, USA).

### 2.8. Explorer and JAK/STAT Phospho-Antibody Arrays

To evaluate SKD-induced changes in signaling pathways, we first employed the Phospho-Explorer Antibody Array (cat# PEX100, Full Moon Biosystems, Sunnyvale, CA, USA) in MDA-MB-468 cells, selected for their heightened sensitivity to SKD and evidence of caspase-3–mediated apoptosis at nanomolar concentrations. Cells were treated with 50 nM SKD for 6 h, and lysates were prepared and processed according to the manufacturer’s instructions. This platform enabled simultaneous analysis of phosphorylation and total protein levels across 1300 signaling proteins.

To further explore SKD’s impact on a specific regulatory axis, we performed a JAK/STAT Phospho Antibody Array (cat# PJS042, Full Moon Biosystems, Sunnyvale, CA, USA) in both MDA-MB-468 and MDA-MB-231 cell lines. MDA-MB-468 cells were treated with 50 nM SKD, while MDA-MB-231 cells received 75 nM SKD. Following the manufacturer’s protocol, proteins were extracted using a mild, non-denaturing buffer to preserve protein conformation. Extracts were biotinylated and purified, then hybridized onto pre-blocked array slides to minimize background and ensure specificity of detection.

Following incubation with the protein samples, the arrays were washed extensively and incubated with Cy3-conjugated streptavidin dye for detection. After further washing to remove unbound dye, the arrays were scanned at a resolution of 10 µm using a compatible microarray scanner. This array enabled simultaneous detection and analysis of phosphorylated JAK/STAT pathway proteins, allowing assessment of pathway activation in response to SKD treatment. Image J was used to quantify the intensity of each well.

### 2.9. Proteomics & Ingenuity Pathway Analysis (IPA)

To identify differentially expressed proteins following Simalikalactone D (SKD) treatment, tandem mass tag (TMT)-based quantitative proteomics was performed on protein lysates from TNBC cell lines MDA-MB-231 and MDA-MB-468. Cells were treated with SKD or vehicle control, and lysates were prepared from ≥4 biological replicates per condition. Proteomics sample preparation and mass spectrometry analysis were performed at the Translational Proteomics Center (TPC) of the University of Puerto Rico Medical Sciences Campus, following optimized protocols described in published literature [21,22,23,24,25]. Briefly, protein concentrations were determined using a Pierce™ 660 nm Protein Assay Kit (cat# 22662, Thermo Fisher, Waltham, MA, USA) and equal amounts of protein from each sample were subjected to reduction with 1,4-dithiothreitol, alkylation with 10 mM iodoacetamide in 50 mM ammonium bicarbonate, and overnight digestion with trypsin using a 1:50 trypsin: protein ratio. Peptides were labeled with TMT 11-plex reagents (cat# A34808, Thermo Fisher, Waltham, MA, USA) according to the manufacturer’s instructions. Labeled peptides were then pooled and fractionated using Pierce high pH reversed-Phase Peptide Fractionation Kit (cat# 84868, Thermo Fisher, Waltham, MA, USA) following the manufacturer’s instructions before analysis using liquid chromatography–tandem mass spectrometry (LC-MS/MS) on an Easy-nLC 1200 system coupled to a Q Exactive Plus Orbitrap mass spectrometer (Thermo Fisher, Waltham, MA, USA). Raw spectra were processed using Proteome Discoverer version 2.1 Thermo Fisher, Waltham, MA, USA). Peptide–spectrum matches (PSMs) were identified with the Sequest HT search engine against the UniProt (Universal Protein Resource, © 2002–2025 UniProt Consortium) human proteome database.

Quantitative analysis of TMT data was performed using the MSstatsTMT R package 2.16.0 [26]. The peptide–spectrum match (PSM) report generated by Proteome Discoverer 2.1 was processed and formatted according to MSstatsTMT guidelines. Shared peptides and low-confidence identifications were removed before analysis. Protein-level summarization was performed using the median polish method, which aggregates peptide-level intensities into a single abundance value per protein per condition. Data were normalized using global median normalization across all TMT channels to correct for systematic variation. Differential protein abundance between SKD-treated and control conditions was evaluated independently for each cell line using linear models with empirical Bayes variance moderation. Proteins were considered significantly differentially expressed if they had a raw *p*-value < 0.05 and an absolute log_2_ fold change ≥ 0.5.

### 2.10. Clustering and Network Analysis

To determine the functional networks and pathways associated with the differentially expressed proteins identified through TMT labeling, Ingenuity Pathway Analysis (IPA) (Ingenuity Systems, Qiagen, Redwood City, CA, USA) was conducted. Expression log_2_ fold change |>0.5| was selected as the measurement type, and a *p*-value cutoff of ≤0.05 was applied to define significant proteins in the IPA Core analysis. The analysis identified the top 30 most relevant pathways for each cell line, MDA-MB-468 and MDA-MB-231.

### 2.11. Computational Analyses and Molecular Docking of SKD

Molecular docking analyses were conducted for the interaction of SKD with STAT4 using DockThor [27,28]. The PDB structures of the target protein were obtained from the RCSB Protein Data Bank based on their relevance and structural resolution [29]. SKD’s molecular structure was retrieved from PubChem and downloaded in SDF format [30]. To ensure compatibility with docking software, Open Babel was used to convert the molecule into the required formats [31].

The docking simulations were configured with parameters of 2 cluster conformers and 3 binding modes per protein-ligand complex. After uploading the prepared protein and ligand files to DockThor, the docking process was carried out, which took approximately 30 min for completion. Further analyses and images were created using ChimeraX [32]. To evaluate the pharmacokinetic properties of SKD, its SMILES representation was analyzed through SwissADME, providing insights into its drug-likeness and ADME profiles [33].

### 2.12. Statistical Analysis

All experiments were conducted in triplicate at minimum, and data were analyzed using GraphPad Prism 10.6.1 software (GraphPad Software, La Jolla, CA, USA). Statistical significance was determined using two-tailed, unpaired Student’s t-tests for comparisons between two groups, and two-way ANOVA for multiple group comparisons, as appropriate for the experimental design. Significance levels were indicated as follows: *p* ≤ 0.05 (*), *p* ≤ 0.01 (**), *p* ≤ 0.001 (***), and *p* ≤ 0.0001 (****). A *p*-value of less than 0.05 was considered statistically significant.

## 3. Results

### 3.1. MDA-MB-468, MDA-MB-231 and SUM-149 Exhibited Different Sensitivity to SKD Treatment

**Figure 1:**
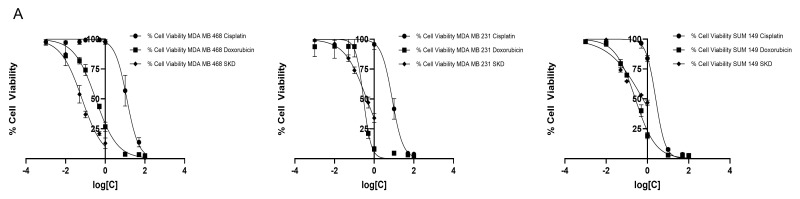

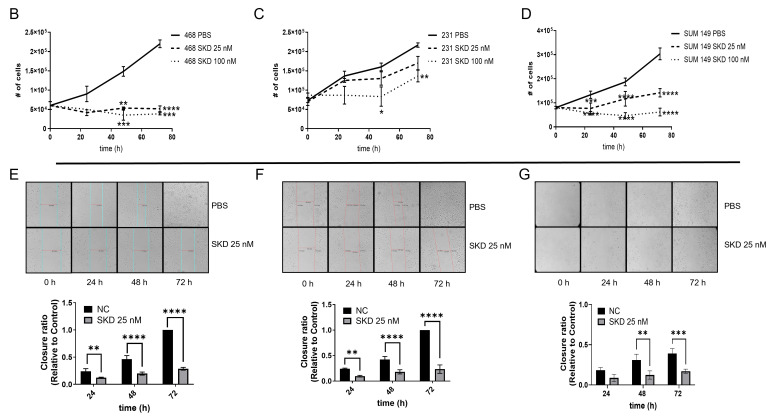
Cytotoxic effect of SKD in MDA-MB-231, MDA-MB-468 and SUM-149 cells. (**A**) Dose–response curves for MDA-MB-231 MDA-MB-468 and SUM-149 cells treated with Doxorubicin, Cisplatin or SKD. (**B**–**D**) Proliferation rates of MDA-MB-468, MDA-MB-231 and SUM-149 cells at 24, 48, and 72 h following treatment with 25 nM and 100 nM SKD. Wound healing assay in (**E**) MDA-MB-231, (**F**) MDA-MB-468, (**G**) SUM-149 following SKD treatment. Cells were treated with SKD 25 nM for 72 h. Bars: triplicates +/− SD. (* *p* < 0.05, ** *p* < 0.01, *** *p* < 0.001, **** *p* < 0.0001).

To evaluate the cytotoxic effects of SKD in TNBC, cell viability was assessed in MDA-MB-468, MDA-MB-231, and SUM-149 cells using the Alamar Blue assay. Cells were treated with increasing concentrations of SKD (1–1000 nM) for 72 h, resulting in a dose-dependent decrease in viability across all models. MDA-MB-468 cells were the most sensitive, displaying an IC_50_ of 67 nM, followed by MDA-MB-231 (IC_50_ = 422 nM) and SUM-149 (IC_50_ = 598 nM) (Figure 1A).

For comparison, the cytotoxic effects of the first-line chemotherapeutics doxorubicin and cisplatin were also quantified in the three TNBC cell lines using a similar assay and a concentration range of 0.001–100 μM (Figure 1A).

Proliferation curves further supported these results. In MDA-MB-468 cells, SKD significantly inhibited proliferation at both 25 nM and 100 nM across all time points (24, 48, and 72 h) (** *p* < 0.01, *** *p* < 0.001, **** *p* < 0.0001) (Figure 1B). In contrast, MDA-MB-231 cells showed significant impairment only at 100 nM and with a more modest effect (* *p* < 0.05, ** *p* < 0.01) (Figure 1C). In SUM-149 cells, both doses significantly reduced proliferation at each time point, although to a lesser extent than in MDA-MB-468 (*** *p* < 0.001, **** *p* < 0.0001) (Figure 1D). These findings suggest that molecular differences may contribute to the different sensitivity of each cell line to SKD treatment.

### 3.2. Low Concentration of SKD Inhibits Cell Migration in MDA-MB-468, MDA-MB-231 and SUM-149 Cells

Although MDA-MB-231 was less sensitive to SKD than MDA-MB-428, MDA-MB-231 is phenotypically characterized by its highly invasive and migratory capacity [16,34]. Therefore, we evaluated whether low-dose SKD (25 nM) could impair cell motility. SKD significantly reduced migration in all three TNBC models compared with untreated controls (Figure 1E–G). These effects became more pronounced at 72 h, when untreated MDA-MB-468 and MDA-MB-231 cells achieved complete wound closure (~100%), whereas SKD-treated cells closed less than 25% of the wound. In SUM-149 cells, full closure was not reached by either group, yet SKD still significantly reduced wound closure relative to the control.

### 3.3. SKD Induces Apoptosis in MDA-MB-468 Cells

To investigate whether SKD-induced cytotoxicity involves apoptotic mechanisms, we measured caspase-3 activity and analyzed the expression of key apoptotic proteins.

**Figure 2:**
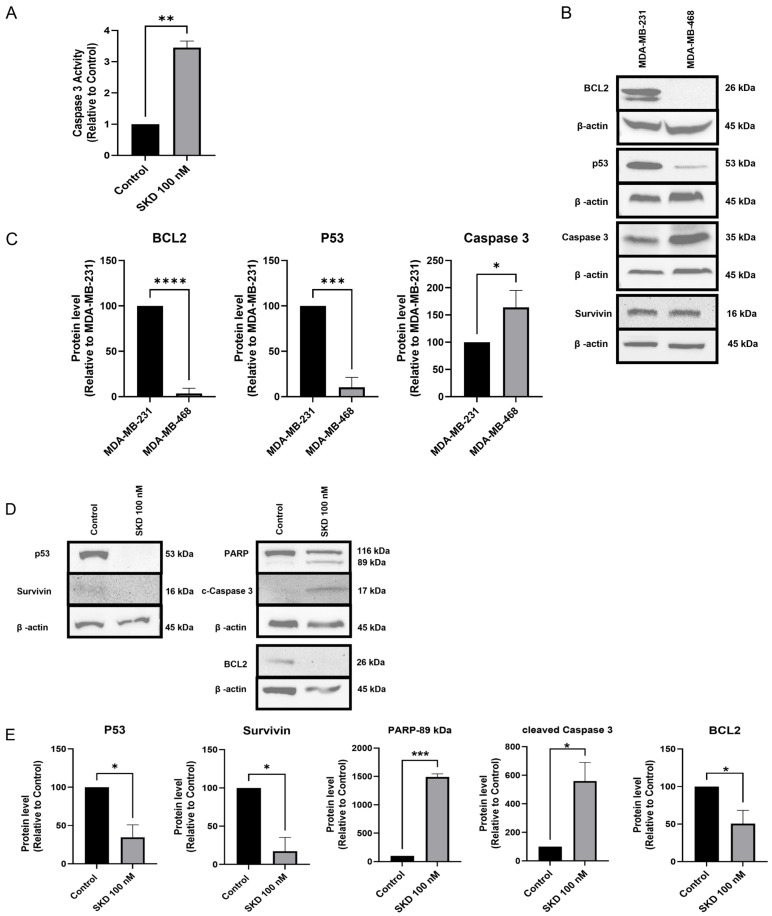
Effect of SKD on apoptosis in TNBC cells. (**A**) MDA-MB-468 cells (4.5 × 10^4^) were exposed to 100 nM SKD for 48 h. Caspase 3 activity was measured as described in the Section 2. (**B**) Representative Western blot images of apoptotic/anti-apoptotic protein markers showing basal levels in MDA-MB-231 and MDA-MB-468 cells and (**C**) quantification by densitometric analysis. (**D**) Representative Western blot images showing the changes in apoptotic-related proteins following a 48 h incubation of MDA-MB-468 cells with 100 nM SKD, and (**E**) densitometric analysis of the band intensities of the Western blot images. Experiments were performed in triplicate. Bars: triplicates +/− SD. (* *p* < 0.05, ** *p* < 0.01, *** *p* < 0.001, **** p < 0.0001).

Treatment of MDA-MB-468 cells with 100 nM SKD for 48 h resulted in a significant 3-fold increase in caspase-3 activity compared to untreated cells (Figure 2A). Western blots confirmed that MDA-MB-468 cells express higher amounts of caspase-3 compared to MDA-MB-231 cells (Figure 2B,C). However, MDA-MB-231 exhibits higher amounts of GOF p53 (which is endowed with anti-apoptotic features, [35]) and anti-apoptotic BCL-2 as compared to MDA-MB-468 cells (Figure 2B,C). The protein levels of survivin, another anti-apoptotic protein, were similar in both cell lines.

Treatment with 100 nM SKD confirmed activation of the apoptotic pathway in MDA-MB-468, as evidenced by a significant increase in cleaved caspase-3 (*p* < 0.05) and cleaved PARP-1 (*p* < 0.001) (Figure 2D,E). As expected, this dose did not induce visible apoptotic features in MDA-MB-231 cells (Supplementary Figure S2). Figure 2D,E confirmed that GOF p53, Bcl-2, and survivin are in the SKD-activated apoptotic pathway of MDA-MB-468 cells as their protein levels were reduced following SKD treatment.

### 3.4. SKD Modulates EGFR and JAK–STAT Signaling

**Figure 3:**
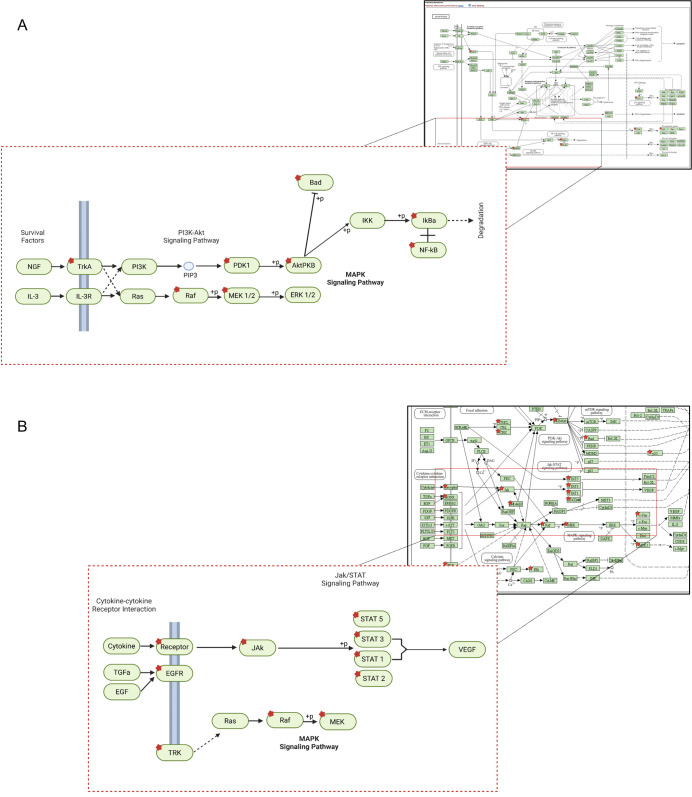
Molecular pathway analysis of the phospho antibody explorer array results. Diagrams show the most relevant molecular pathways altered following treatment of MDA-MB-468 with 50 nM SKD for 6 h. (**A**) Decreased levels of phosphorylated proteins (red stars) of the apoptosis pathway. (**B**) Decreased levels of total proteins supporting modulation of JAK/STAT signaling pathway. Pathways were generated using davidbioinformatics.nih.gov.

As MDA-MB-468 cells are highly sensitive to SKD treatment, we next investigated the early molecular events triggered by SKD in these cells, focusing on changes in protein phosphorylation levels involved in multiple signaling pathways following SKD treatment. For this purpose, we used a Phospho-Explorer Antibody Array consisting of 1318 site-specific antibodies (each in duplicate) from over 30 signaling pathways. Treatment with 50 nM SKD for 6 h led to a ≥25% decrease in the expression levels of 103 out of 1318 site-specific and phospho-specific antibodies (Supplementary Figure S3). Table 1 includes the top five (phospho) proteins whose levels were reduced following SKD treatment. A diagram generated by davidbioinformatics.nih.gov explains the affected targets of the apoptosis pathway (Figure 3A). Notably, several apoptosis-related proteins—including p53, BAD, DAXX, AKT1, and JUN—were significantly decreased upon SKD treatment (Figure 3A). In addition, we observed reduced expression of multiple protein members of the JAK/STAT signaling pathway, including STAT1, STAT2, STAT3, STAT5A, JAK2, AKT1, EGFR, and IL7R (Figure 3B). "Pathways in Cancer" analysis from davidbioinformatics.nih.gov is shown in Supplementary Figure S4, highlighting the JAK/STAT altered proteins (red dots) after SKD treatment in MDA-MB-468 cells, suggesting early disruption or degradation of the pathway’s signaling machinery. Given the number of altered proteins within this pathway following SKD treatment, we further used a specific JAK/STAT phospho-specific antibody array. This array contains 21 distinct phosphorylated proteins and 21 non-phosphorylated counterpart versions. Three antibodies—STAT1 (Ab-701), STAT3 (Ab-705), and STAT5A (Ab-694)—were found in both the Explorer and JAK/STAT-specific phospho-antibody arrays, enabling direct comparison of their expression levels following SKD treatment. These overlapping targets provide consistency between platforms and help validate the observed modulation of JAK/STAT pathway components. The inclusion of multiple phosphorylation-specific variants of these STAT proteins in the JAK/STAT array further enriched the resolution of their post-translational regulation under SKD exposure. Incubation of MDA-MB-468 cells with 50 nM SKD for 6 h led to a reduction in the phosphorylation levels of four proteins in more than 15% (Supplementary Figure S5). Table 2 shows the top three proteins with the most marked reductions in phosphorylation levels upon SKD treatment in each cell line. Particularly, the p-STAT4 (Y693), p-STAT6 (T645), and p-TYK2 (Y1054) in MDA-MB-468 cells and p-STAT4 (Y693), p-JAK2 (T1007), and p-MEK1 (S221) in MDA-MB-231 cells exhibited the greatest decreases in targets analyzed.

These findings suggest that these proteins may be direct targets of SKD or represent early components of signaling pathways leading to apoptosis. Although low doses of SKD did not significantly inhibit proliferation in MDA-MB-231 cells, they did impair cell migration. To assess whether SKD modulated similar phosphoproteins in this context, we analyzed phosphorylation changes in MDA-MB-231 following 6h incubation with 75 nM SKD treatment (Supplementary Figure S6). Table 2 shows the proteins with major changes in their phosphorylation levels following SKD treatment. Notably, we observed a significant decrease in p-STAT4 (Y693) and p-JAK2 (Y1007), and a modest reduction in p-MEK1(S221) was also detected, though this effect was less pronounced (Supplementary Figure S6). The partial overlap in STAT4 modulation suggests that SKD targets the JAK/STAT axis in both TNBC subtypes, with STAT4 emerging as a potential common effector (Supplementary Figures S5 and S6). These findings point to early modulation of the JAK/STAT pathway by SKD, with more pronounced and broader inhibition in the EGFR-high MDA-MB-468 cells (Table 2).

### 3.5. Computational Docking Supports SKD Interaction with STAT4 and EGFR

Phospho-antibody array analysis showed that SKD markedly reduced phosphorylation of STAT4 at Y693 in both MDA-MB-468 and MDA-MB-231 cells, suggesting a potential disruption of STAT4 activation. Because EGFR is an upstream regulator of STAT signaling and is more abundantly expressed in MDA-MB-468 and SUM-149 cells compared with MDA-MB-231 [11,36], we hypothesized that SKD may also interact with EGFR. This could explain the heightened sensitivity of MDA-MB-468 and the enhanced growth-inhibitory response observed in SUM-149 following SKD treatment [36,37]. To explore these possibilities, we performed molecular docking simulations using the DockThor web server [27,28], evaluating SKD’s binding affinity to both STAT4 and EGFR.

**Figure 4:**
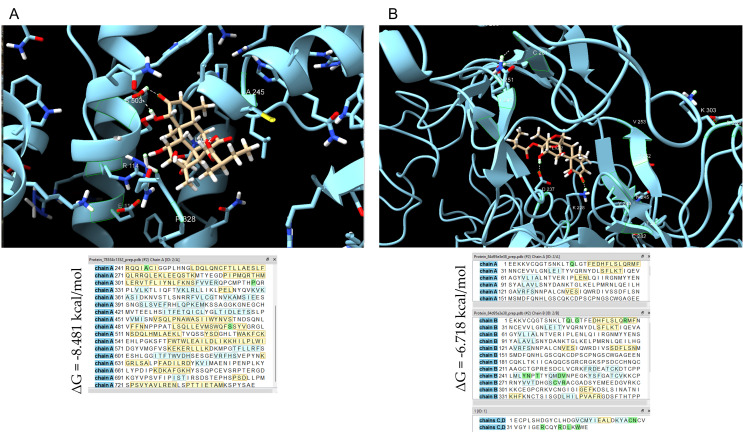
Computational modeling. (**A**) Molecular docking analysis showing SKD’s binding affinity with STAT4 (−8.481 kcal/mol) and (**B**) Binding affinity of SKD with EGFR (−6.718 kcal/mol). Green residues indicate atomic interactions where the center-to-center distance is ≤2.0 Å, excluding interactions between residues fewer than five positions apart in the primary sequence, using the “*include intermodel*” parameter to identify contacts between atoms of two separate structures. Blue regions represent β-sheets, yellow regions correspond to α-helices, and white regions (uncolored) denote coils or loops.

SKD exhibited a binding affinity to STAT4 of –8.481 kcal/mol (Figure 4A), indicating a strong and energetically favorable interaction. However, SKD did not bind at the phosphorylation site (Y693), suggesting that the inhibition of STAT4 phosphorylation may be mediated indirectly or through allosteric effects. Docking analysis with EGFR yielded a binding affinity of −6.718 kcal/mol (Figure 4B), also suggesting a favorable interaction. In both cases, the docking results showed negative van der Waals and electrostatic interaction energies (Table 3), supporting the formation of stable SKD–protein complexes [38].

### 3.6. Proteomic Profiling Reveals Cell-Specific Disruption of Apoptosis and Migration Pathways

**Figure 5:**
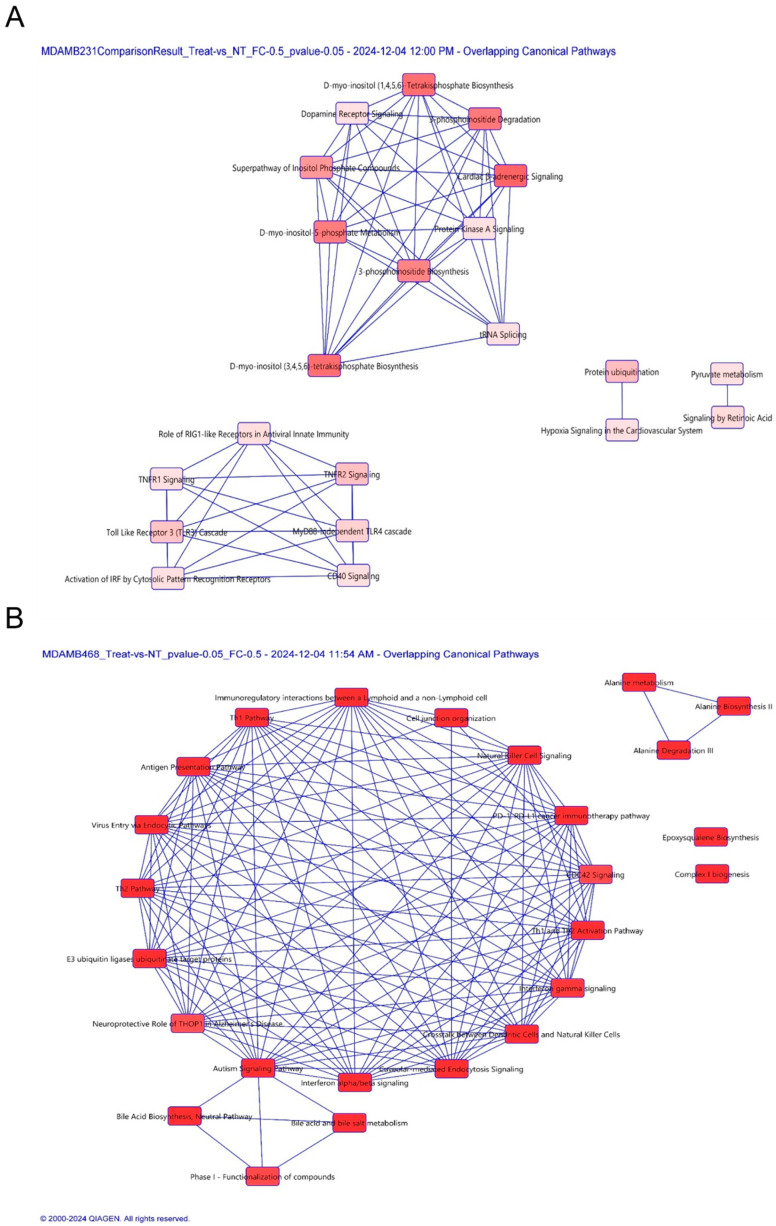

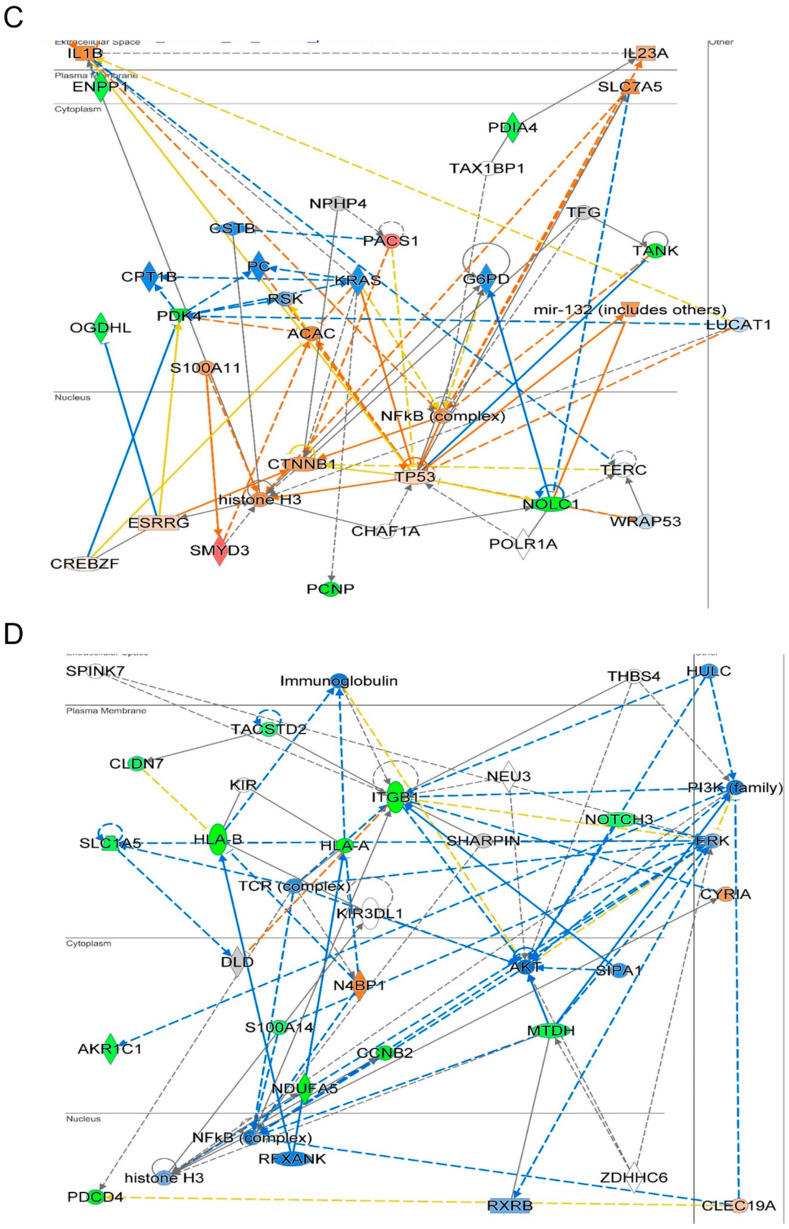

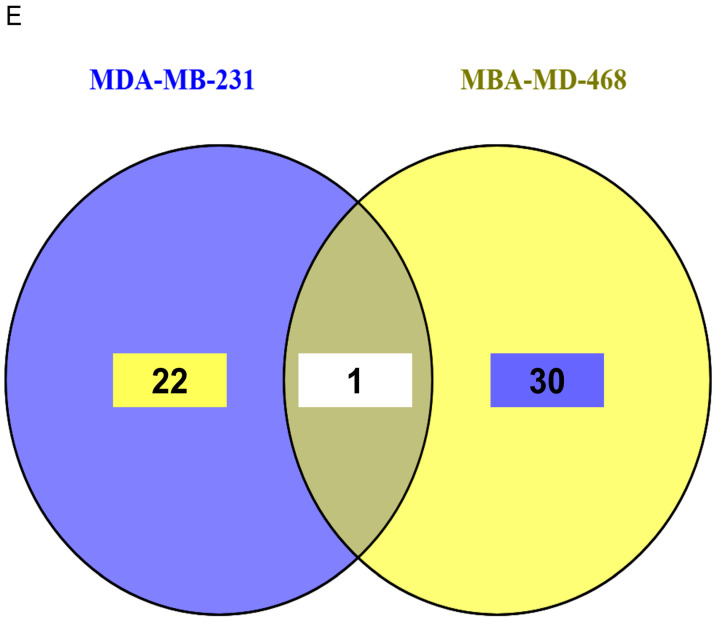
IPA of the proteomic studies. (**A**) IPA of the top 30 canonical pathways altered by SKD in MDA-MB-231 cells, (**B**) IPA of the top 30 canonical pathways impacted by SKD in MDA-MB-468 cells, (**C**) Network displaying relationships among dysregulated proteins in MDA-MB-231 cells, (**D**) Network displaying relationships among dysregulated proteins in MDA-MB-468 cells, (**E**) Venn diagram showing that only ITGB1 protein levels were decreased in both cell lines.

To further investigate changes (48 h following drug treatment) in the proteome following SKD treatment of MDA-MB-468 and MDA-MB-231 cells with SKD, we performed quantitative proteomic analysis using Tandem Mass Tag (TMT) Spectroscopy. Proteins with a *p* ≤ 0.05 and |log_2_ fold change| ≥ 0.5 were considered statistically significant. This filter yielded 31 dysregulated proteins in MDA-MB-468 cells (3 upregulated, 28 downregulated) and 23 in MDA-MB-231 cells (8 upregulated, 15 downregulated) compared with their respective controls (untreated cells). Pathway enrichment analysis using Ingenuity Pathway Analysis (IPA) identified over 30 canonical pathways altered by SKD in both cell lines (Figure 5A–E; Table 4). In MDA-MB-468 cells, SKD affected pathways related to E3 ubiquitin ligase function, PD-1/PD-L1 immune signaling, natural killer (NK) cell signaling, and cell junction organization, many of which are involved in apoptosis, immune evasion, and cytoskeletal remodeling. Notably, pro-apoptotic and structural regulators such as PDCD4, CCNB2, ITGB1, and HLA-A were significantly downregulated, while LARP4, ME3, and IQGAP2 were upregulated (Table 5). These changes align with the activation of apoptosis and reduced migration observed in MDA-MB-468 cells [39,40,41]. In contrast, MDA-MB-231 cells, which are non-responsive to low SKD doses, showed SKD-induced disruption in protein ubiquitination, TNFR2 signaling, D-myo-inositol tetrakisphosphate biosynthesis, and PKA signaling networks associated with cellular stress responses, metabolic regulation, and cytoskeletal dynamics and motility (Table 4). Interestingly, Integrin beta-1 (ITGB1) was the only protein significantly decreased in both cell lines, pointing to a shared SKD effect on cell adhesion and migration [42]. This correlates with the migration inhibition observed following SKD treatment (Figure 5E). ITGB1 is a well-established mediator of extracellular matrix adhesion, migration, and invasion across cancer types, including TNBC [43,44]. Its reduced expression has been shown to inhibit migration and invasion in breast cancer models. Therefore, the observed downregulation of ITGB1 in our study may account for SKD’s ability to suppress cell migration in MDA-MB-468, MDA-MB-231 and SUM-149 cells [43,44].

### 3.7. SKD Consistently Reduces ITGB1 Protein Expression in TNBC Models

**Figure 6:**
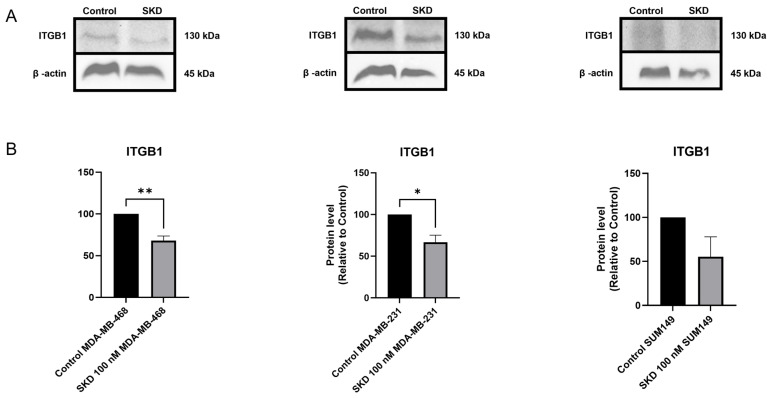
SKD reduces ITGB1 protein levels in TNBC cell lines. (**A**) Representative Western blots and (**B**) densitometric quantification of ITGB1 in MDA-MB-468, MDA-MB-231, and SUM-149 cells treated with 100 nM SKD for 48 h. Experiments were performed in triplicate. Bars: triplicates +/− SD. (* *p* < 0.05, ** *p* < 0.01).

Given the strong migration inhibition observed with SKD and the identification of ITGB1 as a shared target in our proteomic analysis, we next assessed whether SKD reduces ITGB1 protein abundance in TNBC cells. Western blot results confirmed a significant decrease in ITGB1 expression in both MDA-MB-468 and MDA-MB-231 following SKD treatment (Figure 6A,B). Although SUM-149 did not reach statistical significance, a clear downward trend in ITGB1 levels was observed (Figure 6A,B). These data support ITGB1 as a SKD-responsive effector and further suggest that SKD-mediated ITGB1 suppression contributes to reduced motility across distinct TNBC subtypes.

## 4. Discussion

Triple-negative breast cancer (TNBC) represents one of the most aggressive and therapeutically intractable subtypes of breast cancer due to the lack of standard therapeutic targets ER, PR, and HER2 [1,2]. TNBC disproportionately affects younger women and those of African ancestry. It is associated with a high risk of recurrence, metastasis, and resistance to conventional chemotherapy [1,2]. In this study, we investigated the biological activity of SKD, a quassinoid compound isolated from the Puerto Rican endemic tree Simarouba tulae, in MDA-MB-468, MDA-MB-231 and SUM-149, three well-characterized TNBC cell lines with distinct molecular and phenotypic features. All three models are widely used to model inter-tumoral heterogeneity in TNBC and differ in their proliferative signaling, metastatic behavior, subtype, and drug sensitivities [3,4,11,12].

Previous studies have evaluated the cytotoxic potential of SKD in various cancer cell lines [5]; however, to our knowledge, this is the first study to investigate the molecular mechanisms underlying its action in TNBC. Our results suggest that SKD induces its anticancer effects by activating different cellular pathways in MDA-MB-468 and possibly in SUM-149 than in MDA-MB-231. This hypothesis aligns with literature reports showing that MDA-MB-468 and SUM-149 cells are highly dependent on EGFR signaling for survival [36,72]. In contrast, MDA-MB-231 cells are more mesenchymal and rely on alternative signaling axes, including NF-κB and PI3K/AKT, which may confer reduced apoptotic sensitivity at lower drug concentrations of SKD [73,74,75,76]. As noted by other research groups, drug response profiles often fail to correlate directly with genetic or transcriptional features, suggesting that functional profiling offers a more powerful means of identifying context-specific vulnerabilities that can be targeted therapeutically [77]. Furthermore, combining cytotoxicity assessment with molecular pathway analysis provides a more comprehensive understanding of drug action, surpassing traditional viability-based assays and potentially offering more translationally relevant insights [77]. Together, these findings emphasize the importance of understanding genotype-specific vulnerabilities and signaling dependencies to predict therapeutic responses and tailor interventions effectively across heterogeneous TNBC subtypes.

Our findings align with previous reports demonstrating that molecularly distinct breast cancer cell lines exhibit differential responses to the same compound, both phenotypically and mechanistically [78]. For instance, a study evaluating the effects of a phenolic extract showed that while both MCF7 and MDA-MB-231 cells experienced reduced proliferation, only MCF7 cells underwent apoptosis, marked by PARP-1 cleavage, accumulation in sub-G1 phase, and activation of the H2AX signaling pathway [78]. In contrast, MDA-MB-231 cells primarily showed PI3K pathway activation and modest lipid peroxidation, without triggering apoptotic markers [78]. These divergent outcomes can be explained by inherent biological differences between the TNBC models, including receptor signaling dependencies and p53 mutational background. Consistent with this, SKD triggered caspase-3–mediated apoptosis in MDA-MB-468 cells, which overexpress EGFR and lack functional PTEN. In contrast, MDA-MB-231 cells, characterized by KRAS and BRAF mutations and a mesenchymal highly invasive phenotype, exhibited primarily non-apoptotic responses, with reduced motility rather than pronounced cell death. SUM-149 cells, which are also strongly dependent on EGFR signaling [79], displayed significant suppression of proliferation as early as 24 h following treatment, even at sublethal SKD concentrations, along with diminished migratory capacity. These contrasting outcomes may also be attributed to differences in their basal apoptotic machinery. MDA-MB-468 cells exhibit higher basal levels of caspase-3 and lower expression of anti-apoptotic proteins like BCL-2 and are more primed for apoptosis compared to MDA-MB-231 cells. In contrast, MDA-MB-231 cells are more resistant to apoptosis, possibly due to GOF mutations in p53, which are known to promote a therapy-resistant phenotype by enhancing survival pathways and inhibiting apoptotic responses [80], and is more elevated in MDA-MB-231 cells than in MDA-MB-468 cells. This inherent difference in apoptotic readiness likely contributes to the observed incongruence in SKD-induced cell death between the two models.

Phosphoarray data further revealed cell-specific alterations in the JAK/STAT signaling cascade. In MDA-MB-468 cells, SKD reduced phosphorylation of EGFR downstream targets, including p-STAT3, p-STAT4, and p-STAT5—consistent with apoptosis [37,81]. Liao et al. previously showed that STAT3 activation promotes TNBC survival by upregulating anti-apoptotic proteins such as BCL-2 and Mcl-1. Inhibition of p-STAT3 leads to caspase 3 activation and apoptosis in TNBC models, in agreement with our findings [82]. Also, STAT4 phosphorylation underlies cell survival and proliferation in other cancer types. For instance, prolonged downregulation of p-STAT4 is linked to cytochrome c-mediated caspase-3 activation and apoptotic cell death in hematopoietic and NK cells [83]. Multiple studies have shown that STAT5 activity drives expression of anti-apoptotic factors like BCL-xL. Inhibition of STAT5 phosphorylation reduces Bcl-xL levels and triggers caspase-3-dependent apoptosis in several types of cancers such as leukemia and melanoma [84,85,86]. In MDA-MB-231 cells, SKD also decreased p-STAT4 and p-JAK2, suggesting that STAT4 may represent a shared SKD-sensitive node, even in the absence of cell death. STAT4 dysregulation has been previously implicated in immune signaling, inflammation, and tumor aggressiveness, but its role in TNBC remains underexplored [87,88]. Furthermore, molecular docking simulations supported these observations by showing that SKD binds to STAT4 and EGFR, though not directly at phosphorylation sites. These interactions are energetically stable and consistent with an allosteric modulation model. The stronger binding to STAT4 (−8.48 kcal/mol) suggests it may be a primary target involved in early signaling disruption (6 h).

Interestingly, migration was suppressed in the three cell lines, even at 25 nM SKD concentration. This effect correlates with our proteomic findings, which revealed downregulation of ITGB1 in both MDA-MB-231 and MDA-MB-468. ITGB1 is a central mediator of cell–matrix adhesion and motility, and its inhibition has been associated with reduced invasion and metastasis in TNBC [40,43,44,54,55,56]. For instance, Klahan et al. showed that silencing ITGB1 significantly inhibits migration and invasion in TNBC cell lines MDA-MB-231 and MDA-MB-468, highlighting its critical role in metastatic behavior [89]. In agreement with this evidence, our Western blot validation confirmed reduced ITGB1 levels across all tested TNBC models (MDA-MB-468, MDA-MB-231, and SUM-149) following SKD treatment, supporting the migration-inhibitory effects observed in our assays and reinforcing our proteomic findings. This suggests that SKD’s anti-migratory effects are not contingent on apoptosis but may involve direct modulation of adhesion and cytoskeletal pathways. Additionally, the proteomic profiling reinforced the idea that SKD engages divergent mechanisms in a concentration-dependent manner. In MDA-MB-468 cells, SKD resulted in the downregulation of key pro-survival and immune evasion proteins, including PDCD4, CCNB2, and HLA-A. Notably, PDCD4 is a tumor suppressor that modulates translation and promotes apoptosis; paradoxically, in some cancer contexts, it can support cell survival under stress [52]. Chen et al. showed that PDCD4 deficiency in macrophages can improve lysosome function and enhance their anti-tumor effect to indirectly inhibit tumor growth [90]. CCNB2 is essential for the G2/M transition, and its loss can lead to cell cycle arrest and facilitate apoptosis, consistent with SKD’s antiproliferative effect. CCNB2 suppression has been shown to induce cell cycle arrest, reduce proliferation, and promote apoptosis in multiple cancer models (e.g., hepatocellular carcinoma) [91]. On the other hand, MDA-MB-231 cells, treated with sub-lethal concentrations of SKD, exhibited alterations in proteins involved in metabolism, transcriptional regulation, and ubiquitin signaling. Notably, PCNP (PEST-containing nuclear protein) has been shown to promote proliferation, migration, and invasion in lung adenocarcinoma by activating p-STAT3 and p-STAT5 signaling, while also suppressing apoptosis—suggesting that its downregulation by SKD may contribute to the observed anti-migratory effect in this cell line [45]. Recent insights into metastatic TNBC suggest that immune landscape differences can significantly influence therapeutic response, even in the absence of classical immune-targeting interventions. A study using single-cell RNA sequencing and proteomics demonstrated that peripheral CD33^+^ myeloid cells exhibited divergent transcriptional programs between responders and non-responders to chemo-immunotherapy. In responders, CD33^+^ cells promoted immune activation, while in non-responders, they supported immunosuppressive phenotypes—highlighting the potential role of myeloid-driven immune modulation in therapeutic outcomes [92]. In our study, proteomic profiling similarly revealed immune-related differences between the two TNBC cell lines analyzed. MDA-MB-231 cells, although less sensitive to SKD-induced apoptosis, showed notable changes in immune-modulatory proteins following treatment. Specifically, decreased expression of TANK and ENPP1—both associated with immune evasion and tumor progression—suggests that SKD may attenuate immunosuppressive pathways in these cells [47,49]. Conversely, the upregulation of PPP1R14B, which is linked to increased infiltration of myeloid-derived suppressor cells and worse prognosis in multiple cancers, implies a complex immunological remodeling potentially favoring an aggressive phenotype [62]. These findings support the notion that MDA-MB-231 and MDA-MB-468 differ not only in their molecular profiles but also in their immune-related responses to SKD, reinforcing the importance of immunological context in shaping drug sensitivity across TNBC subtypes.

Taken together, these results support a model in which cells reliant on EGFR-STAT4 signaling are more sensitive to SKD, which exerts apoptotic and anti-proliferative effects at lower nanomolar concentrations. Although molecular docking suggested potential interactions between SKD and STAT4/EGFR, these predicted binding events have not yet been experimentally validated. More precise approaches, including Isothermal Titration Calorimetry or Biolayer Interferometry, could be used in subsequent experiments to directly evaluate SKD binding affinity and clarify its mechanism of action in TNBC models. SKD consistently inhibits motility across TNBC subtypes, likely through ITGB1 suppression and cytoskeletal remodeling, even at sublethal doses. While our proteomic analysis and ITGB1 validation indicate that SKD may influence proteins involved in immune-related pathways, the functional consequences remain to be determined, and additional studies are needed to assess effects on immune cell activity and other immune responses. Finally, evaluation in TNBC mouse xenograft models, including humanized immune systems, and advanced 3D culture systems will help clarify tumor microenvironment influences, immune-mediated effects, and SKD’s translational potential.

## 5. Conclusions

This study provides novel mechanistic insights into the differential responses of three molecularly distinct TNBC models to SKD. Beyond confirming SKD’s cytotoxic and anti-migratory potential, our results emphasize the importance of using diverse TNBC models to capture the complexity of tumor behavior. They also underscore the need to stratify TNBC patients based on molecular profiles to guide more personalized and effective treatments. While further validation in vivo is warranted, this study lays the groundwork for SKD’s continued exploration as a therapeutic candidate in heterogeneous TNBC.

## Figures and Tables

**Table 1:** Top phosphorylated proteins whose levels were reduced following SKD treatment in MDA-MB-468.

Name	Relative Levels
VE-Cadherin (Phospho-Tyr731)	0.3925
MAP3K8/COT (Phospho-Thr290)	0.4743
BTK (Phospho-Tyr223)	0.5395
CrkII (Phospho-Tyr221)	0.5563
IkB-alpha (Phospho-Ser32/36)	0.5578

**Table 2:** Relative Expression Level of Phosphorylated Proteins Identified in the JAK/STAT Phospho-Antibody Array Compared to Untreated Cells.

Cell Line	Name	Relative Levels
MDA-MB-231	STAT4(Phospho-Tyr-693)	0.563
	JAK2(Phospho-Tyr1007)	0.785
	Mek1(Phospho-Ser221)	0.939
MDA-MB-468	STAT4(Phospho-Tyr-693)	0.719
	STAT6(Phospho-Thr-645)	0.800
	TYK2(Phospho-Tyr1054)	0.817

**Table 3:** Molecular Docking of SKD with STAT4 and EGFR.

Protein of Interest	Binding Affinity (kcal/mol)	Vdw Energy (kcal/mol)	Elec. Energy (kcal/mol)
STAT4	−8.481	−24.299	−9.018
EGFR	−6.718	−7.117	−23.382

**Table 4:** Key cancer-associated canonical pathways altered by the treatment MDA-MB-231 and MDA-MB-468 cells with SKD.

Pathway in MDA-MB-231	*p*-value	Number of Proteins	Proteins
Protein ubiquitination	2.39 × 10^−2^	1	UBE2E3
TNFR2	2.46 × 10^−2^	1	TANK
D-myo-inositol (1,4,5,6)-tetrakisphosphate biosynthesis	9.95 × 10^−3^	2	ENPP1, PPP1R14B
Protein Kinase A Signaling	3.99 × 10^−2^	2	ENPP1, PPP1R14B
Pathway in MDA-MB-468	*p*-value	Number of Proteins	Proteins
E3 ubiquitin ligases ubiquitinate target proteins	1.80 × 10^−3^	2	HLA-A, HLA-B
PD-1, PDL-1 immunotherapy pathway	2.24 × 10^−4^	3	HLA-A, HLA-B, PDCD4
Natural Killer Cell Signaling	1.42 × 10^−3^	3	HLA-A, HLA-B, ITGB1
Cell Junction Organization	4.94 × 10^−3^	2	CLDN7, ITGB1

**Table 5:** Top five decreased and top five increased protein levels identified by proteomics analysis.

Protein	Protein Symbol	log_2_ Fold Change	*p*-Value	Biological Role
Decreased Protein Levels in MDA-MB-231				
Q8WW12	PCNP	−0.80167	0.002767	PEST-containing nuclear protein (PCNP) promotes proliferation, migration, and invasion in lung adenocarcinoma cells while inhibiting apoptosis through p-STAT3 and p-STAT5 activation [45].
Q14978	NOLC1	−0.83644	0.025729	Nucleolar and coiled-body phosphoprotein 1 (NOLC1) supports cancer stem cell properties, promoting tumor growth, therapy resistance, and relapse. Its silence in TNBC cells reduces stemness markers (MYC, ALDH) and sphere formation. High NOLC1 levels are linked to poor prognosis, highlighting its potential as a therapeutic target [46].
Q92844	TANK	−0.63767	0.007082	TRAF family member-associated NF-κB activator (TANK) contributes to an immunosuppressive tumor microenvironment and regulates genes involved in cell survival and inflammation. Its overexpression is linked to poor glioma prognosis [47].
Q969T4	UBE2E3	−0.60915	0.034159	Ubiquitin-conjugating enzyme E2 E3 (UBE2E3) interacts with c-Cbl to upregulate EGFR, activating the MAPK pathway and driving tumor growth and progression [48].
P22413	ENPP1	−0.51164	0.035984	Ectonucleotide pyrophosphatase/phosphodiesterase 1 (ENPP1) suppression in HER2Δ16 tumors slows growth and increases immune infiltration, highlighting its role in limiting immune response in these tumors [49].
Decreased Protein Levels in MDA-MB-468				
Q16718	NDUFA5	−0.88316	0.032715	NADH:Ubiquinone Oxidoreductase Subunit A5 (NDUFA5) mutations or downregulation of this gene impairs mitochondrial activity, reducing ATP production and increasing oxidative stress [50].
Q53EL6	PDCD4	−0.94785	0.002245	Programmed Cell Death Protein 4 (PDCD4) is a well-known tumor suppressor gene. Its low expression is associated with Aromatase inhibitor resistance—a drug primarily used in the treatment of hormone receptor-positive breast cancer, especially in postmenopausal women [51,52]. Its low expression is also associated with poor prognosis and reduced disease-free survival in ER-positive, high-grade tumors [52].
O95067	CCNB2	−0.82997	0.025429	Cyclin B2 (CCNB2) is associated with breast cancer and anauxetic dysplasia 1, playing roles in mitotic cell cycle regulation and microtubule organization [53].
P05556	ITGB1	−0.93412	0.028627	β1 integrin (ITGB1) drives breast tumor progression, supporting proliferation, survival, and resistance to lapatinib. Inhibiting β1 integrin in early-stage breast cancer cells reverses malignant behavior in laminin-rich extracellular matrix cultures [54,55,56].
P04439	HLA-A	−0.87185	0.026263	Human Leukocyte Antigen A (HLA-A) plays a key role in the immune system by encoding proteins that present peptides to T cells, crucial for pathogen defense and identifying abnormal or cancerous cells [57].
Increased Protein Levels in MDA-MB-231				
Q9H7B4	SMYD3	0.536685	0.038703	SET and MYND Domain Containing 3 (SMYD3) upregulation is linked to poor prognosis in cancers by activating oncogenes and promoting cell survival. Its inhibition could suppress tumor growth and enhance chemotherapy sensitivity [58,59].
Q6VY07	PACS1	0.508768	0.046877	Phosphofurin Acidic Cluster Sorting Protein 1 (PACS1) regulates the intrinsic apoptotic pathway by controlling BAX/BAK oligomerization and mitochondrial outer membrane permeabilization. Cells with reduced PACS1 expression resist apoptosis from various stimuli but remain sensitive to TRAIL receptor ligation [60].
Q32P41	TRMT5	0.610423	0.015878	tRNA Methyltransferase 5 (TRMT5) is linked to cancer progression and targeting it may inhibit hepatocellular carcinoma progression and enhance chemotherapy sensitivity [61].
Q96C90	PPP1R14B	0.750876	0.007414	Upregulation of Protein Phosphatase 1 Regulatory Inhibitor Subunit 14B (PPP1R14B) is linked to poor prognosis in cancers, correlating with increased immune cell infiltration, particularly myeloid-derived suppressor cells. This may contribute to an immunosuppressive tumor microenvironment, promoting tumor progression in pancreatic cancer [62].
Q8N6R0	eEF1A-KNMT	0.92339	0.051941	Eukaryotic Translation Elongation Factor 1A Lysine N-Methyltransferase (eEF1A-KNMT) regulates cancer progression by affecting protein synthesis and tumor cell survival. Its overexpression is linked to increased tumor proliferation and metastasis, including triple-negative breast cancer [63,64,65].
Increased Protein Levels in MDA-MB-468				
Q71RC2	LARP4	0.511559	0.008935	La Ribonucleoprotein 4 (LARP4) interacts with mRNAs to regulate cell proliferation by stabilizing transcripts and enhancing their translation. It binds to 3′ UTRs or competes with microRNA machinery [66], and through interaction with PABPC1, promotes synthesis of proteins involved in proliferation and survival [67]. LARP4 is also implicated in survival and metastasis, and its expression decreases during epithelial-to-mesenchymal transition in breast cancer [68,69].
Q16798	ME3	0.679279	0.039963	Malic Enzyme 3, NADP(+)-Dependent, Mitochondrial (ME3), expression is linked to negative lymph node metastasis, and patients with positive ME3 expression have a better prognosis in breast cancer [70].
Q13576	IQGAP2	0.689599	0.053686	IQ Motif Containing GTPase Activating Protein 2 (IQGAP2) may act as a tumor suppressor, with its downregulation linked to poor prognosis in cancers like breast, lung, and gastric. Reduced IQGAP2 expression promotes cell migration, invasion, and EMT, key processes in tumor progression and metastasis [71].

## Data Availability

The original contributions presented in this study are included in the article/Supplementary Material. Further inquiries can be directed to the corresponding authors.

## Supplementary Materials

The following supporting information can be downloaded at: https://www.mdpi.com/article/10.3390/biom15111561/s1, Supplementary Figure S1: The second peak (blue color) was identified by SKD by TLC analysis and NMR. Supplementary Figure S2: Evaluation of the apoptosis pathway in MDA-MB-231 cells. MDA-MB-231 cells (4.5 × 10^4^) were treated with 100 nM SKD for 48 h. Caspase-3 activity was measured as described in the Section 2. Supplementary Figure S3: Explorer phospho-antibody array images. Supplementary Figure S4: Pathways in Cancer from davidbioinformatics.nih.gov. Red dots: decreased protein levels following SKD treatment, 50 nM SKD using MDA MB 468 cell line. Red square showing the JAK/STAT signaling pathway proteins altered. Supplementary Figure S5: JAK/STAT phospho-antibody array images. Supplementary Figure S6: JAK/STAT phospho-antibody array images. Supplementary Figure S7: Western blot images of the complete membranes used to generate Figure 2. Supplementary Figure S8: Western blot uncropped membranes Corresponding to Figure 6.
