# Supplementary material for: Molecular Basis of Simalikalactone D Sensitivity in Triple-Negative Breast Cancer Cells
Source: Biomolecules. 2025 Nov 6;15(11):1561. doi: 10.3390/biom15111561 (PMC12650167; doi:10.3390/biom15111561)

Supplementary Figure S1: Chemical structure and chromatographic profile of Simalikalactone D (SKD).

A

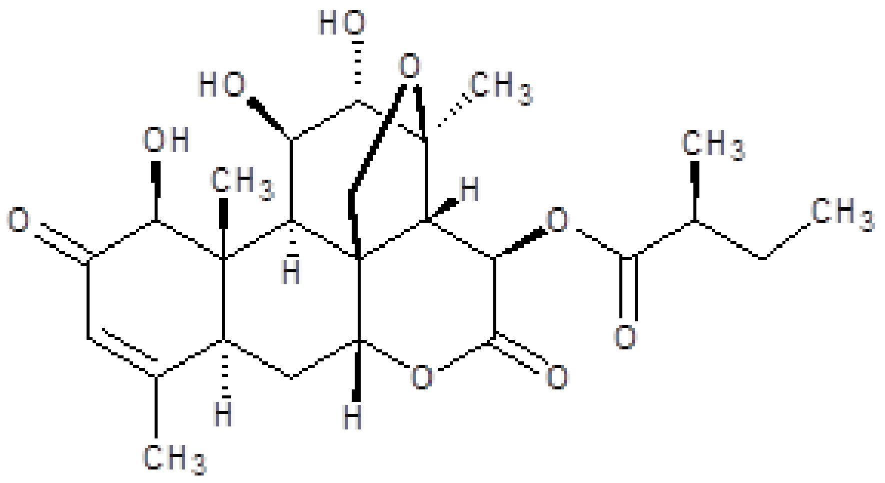

B

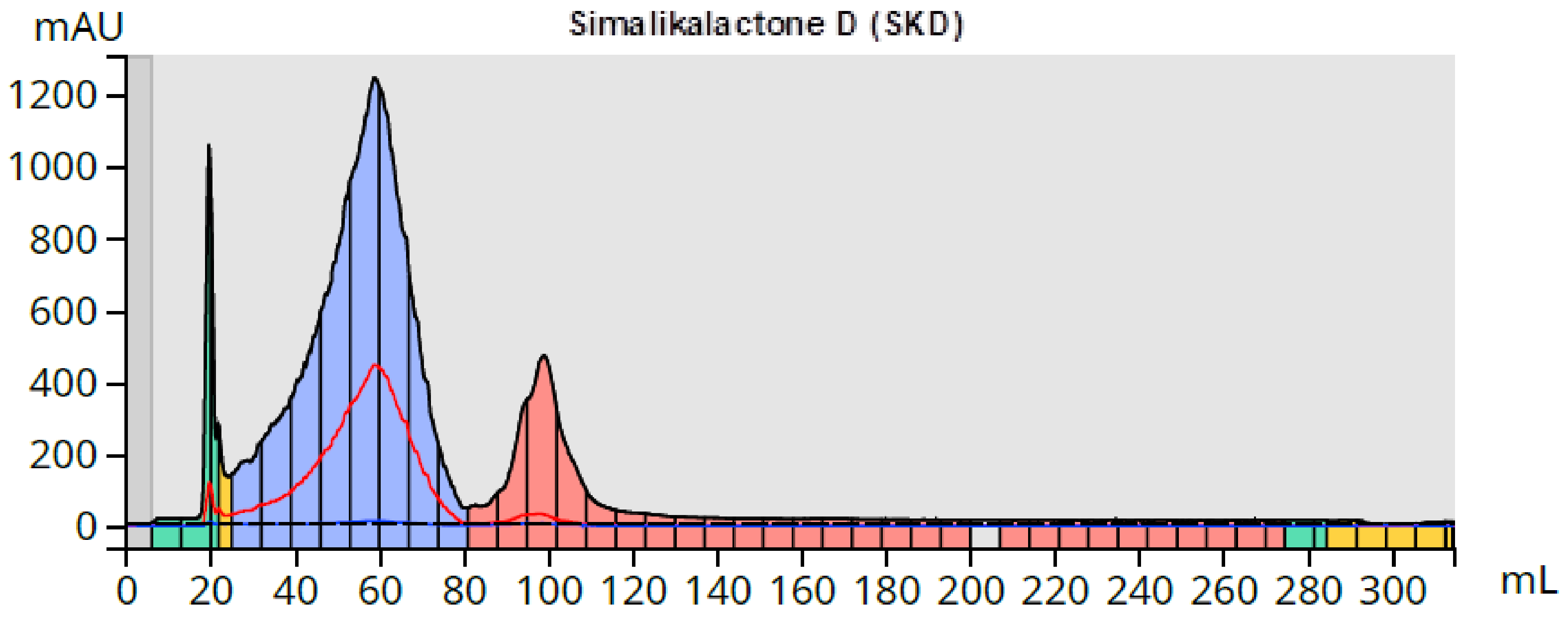

Supplementary Figure S2: Evaluation of apoptosis pathway in MDA-MB-231

A

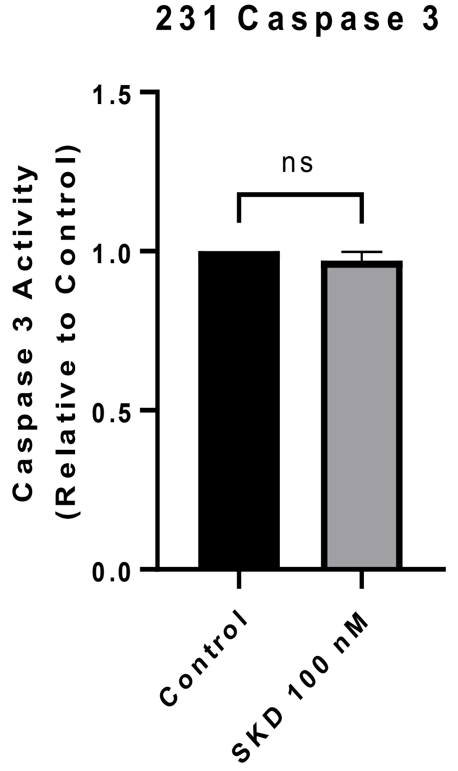

B

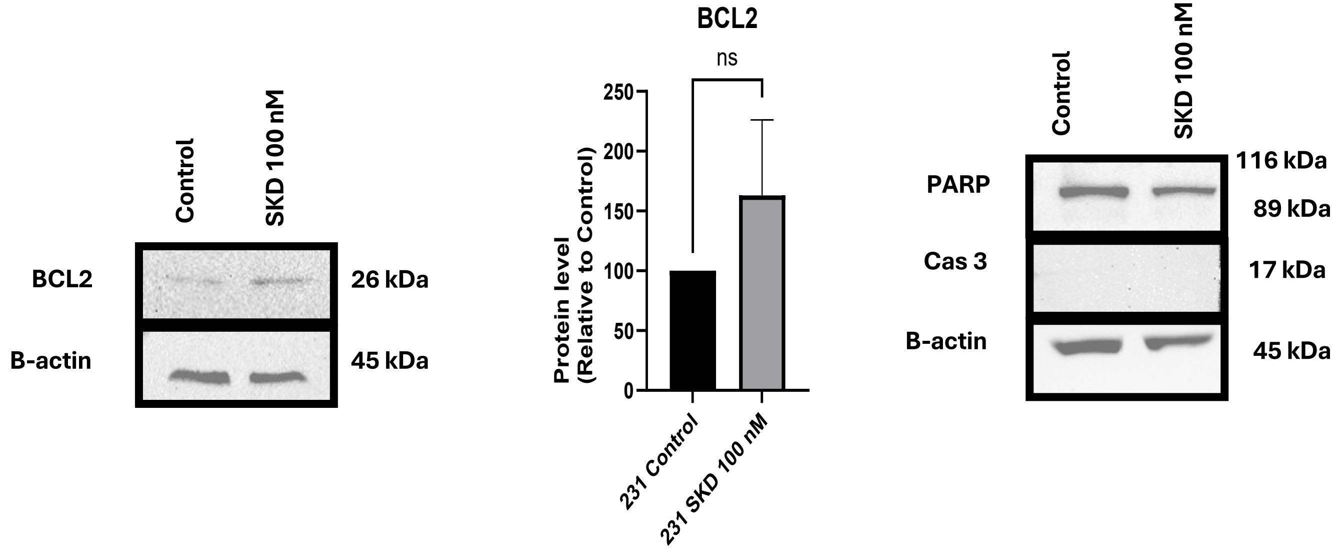

Supplementary Figure S3. Explorer Phospho antibody array after 6h incubation with 50 nM SKD using MDA MB 468 cell line

A

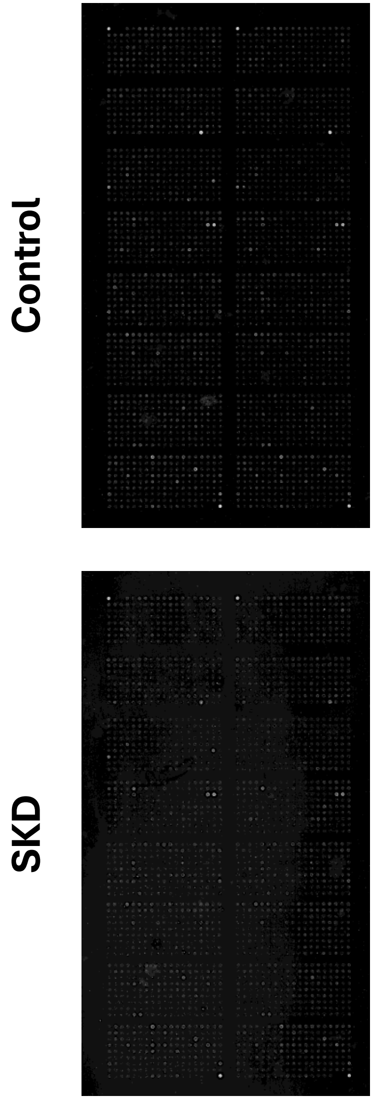

B

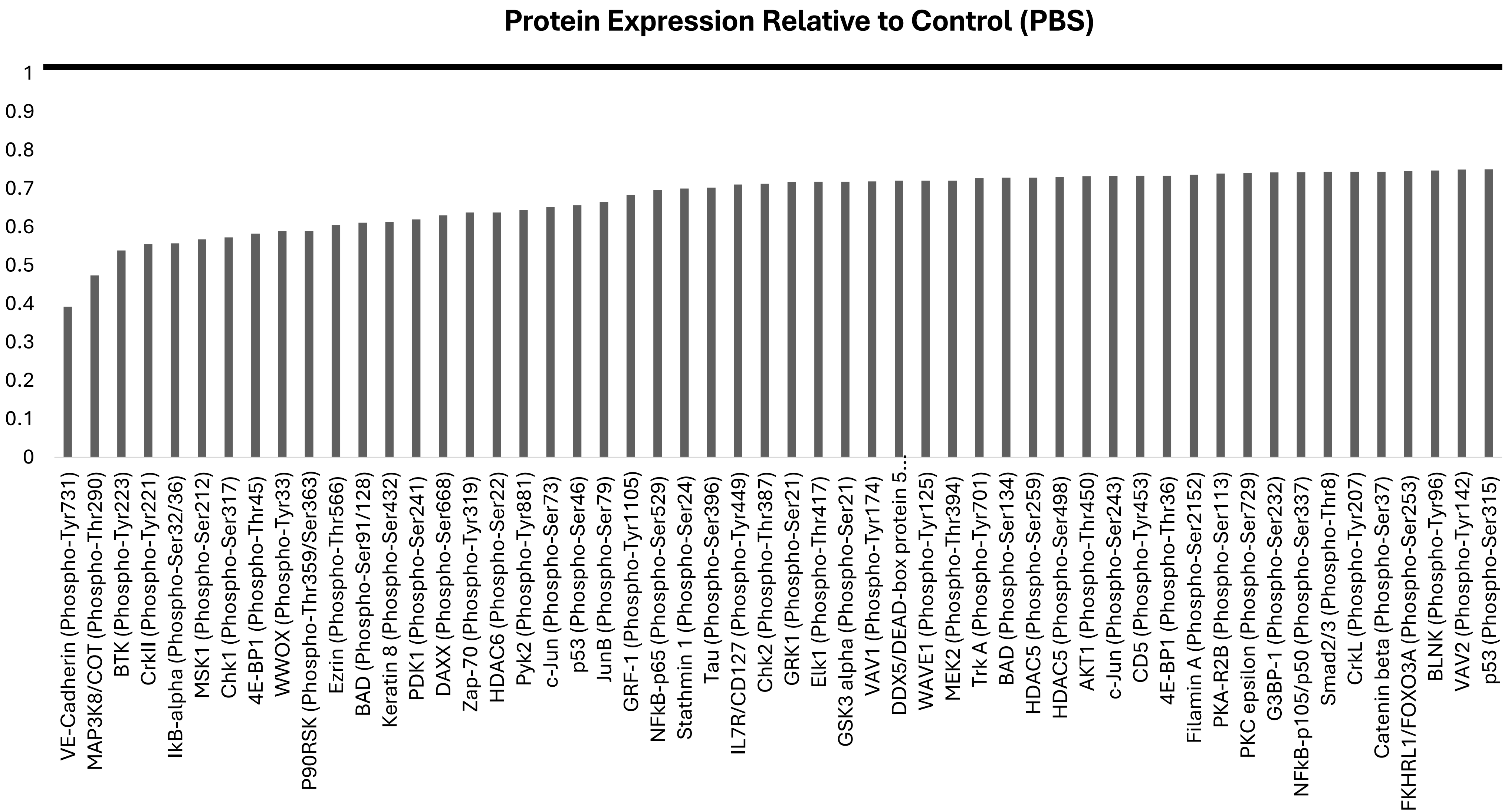

Supplementary Figure S4. Pathways in Cancer from davidbioinformatics.nih.gov. Red dots: decreased protein levels following SKD treatment, 50 nM SKD using MDA MB 468 cell line. Red square showing the JAK/STAT signaling pathway proteins altered.

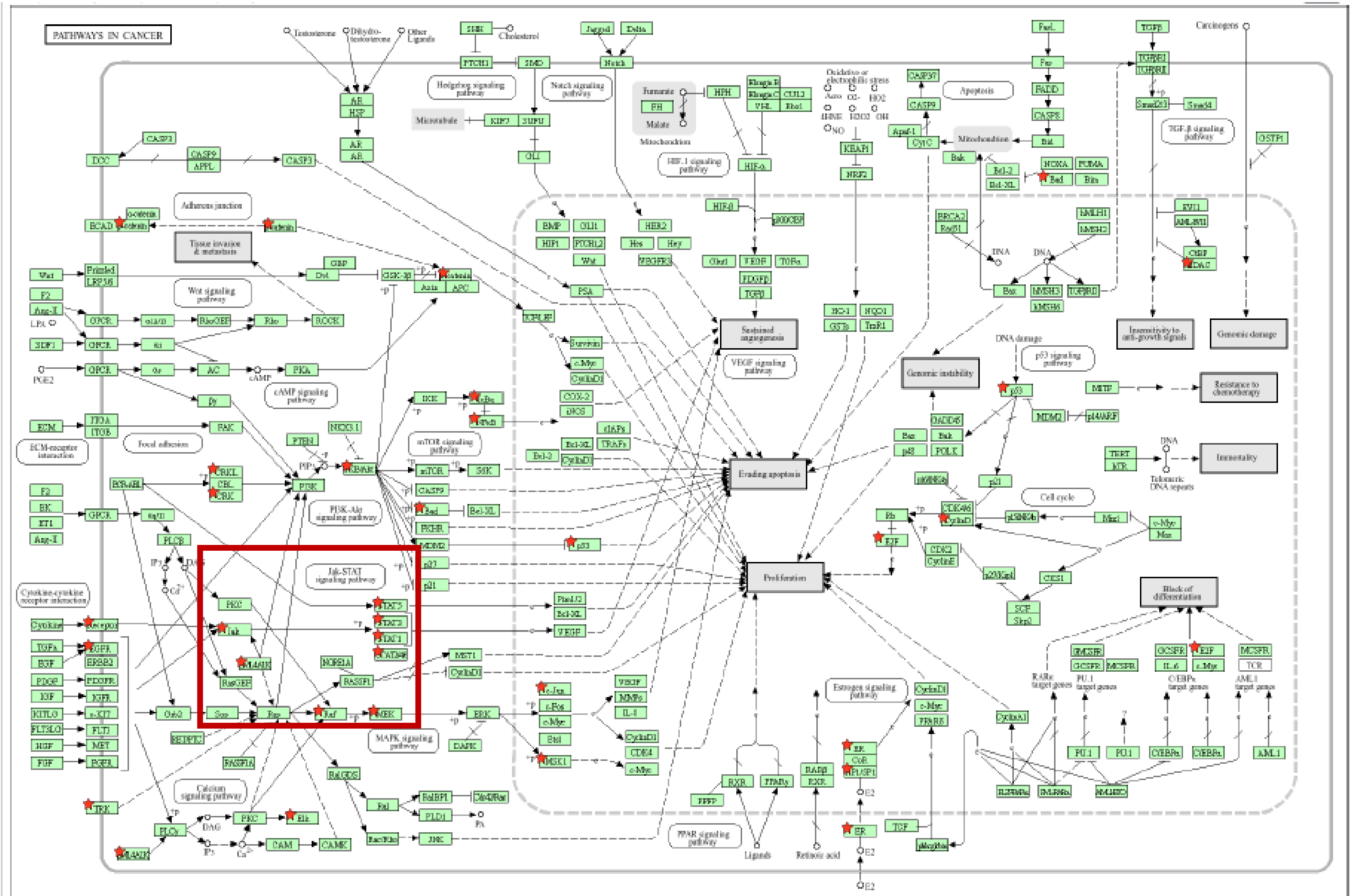

Supplementary Figure S5. JAK/STAT Phospho antibody array after 6h incubation with 50 nM SKD using MDA MB 468 cell line

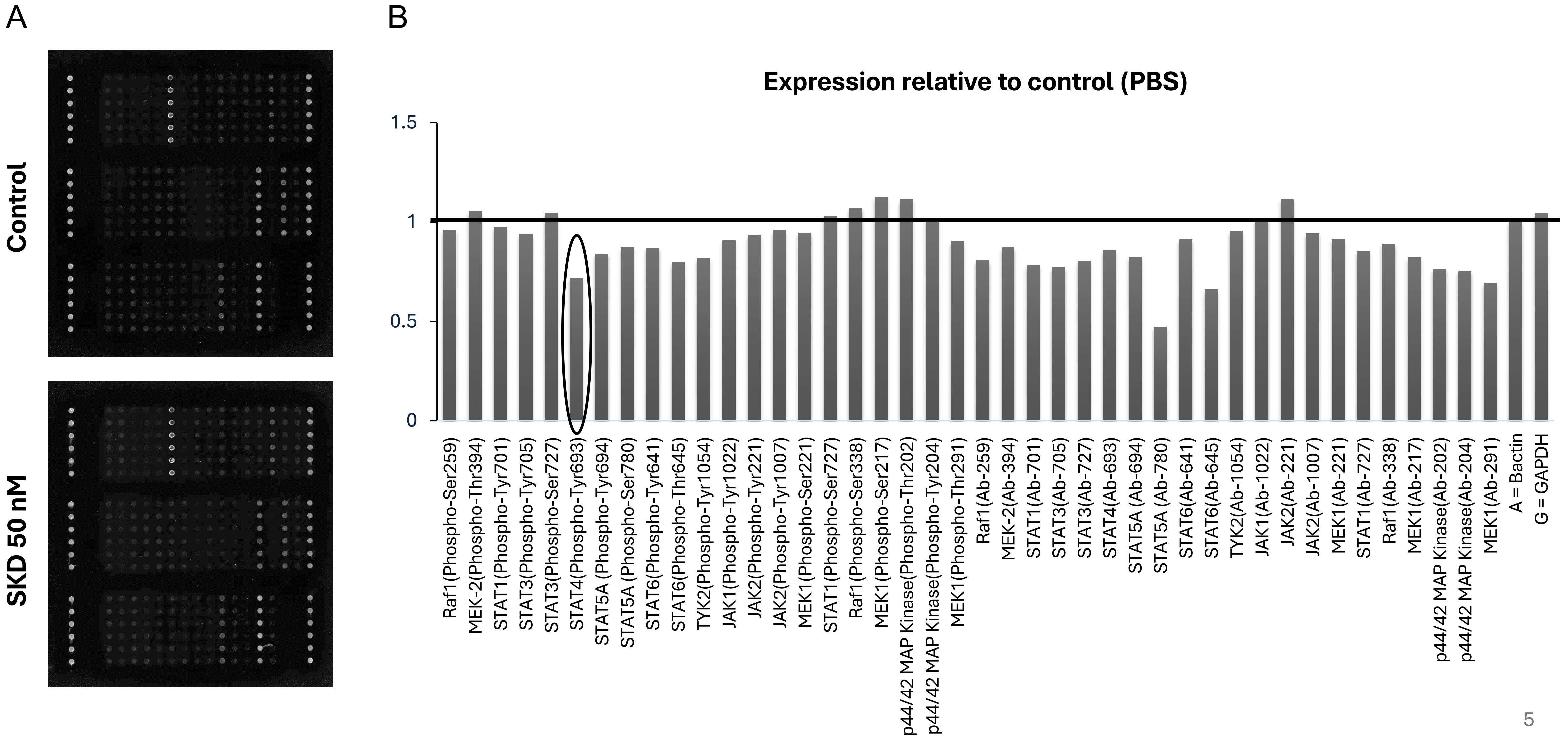

Supplementary Figure S6. JAK/STAT Phospho antibody array after 6h incubation with 75 nM SKD using MDA MB 231 cell line

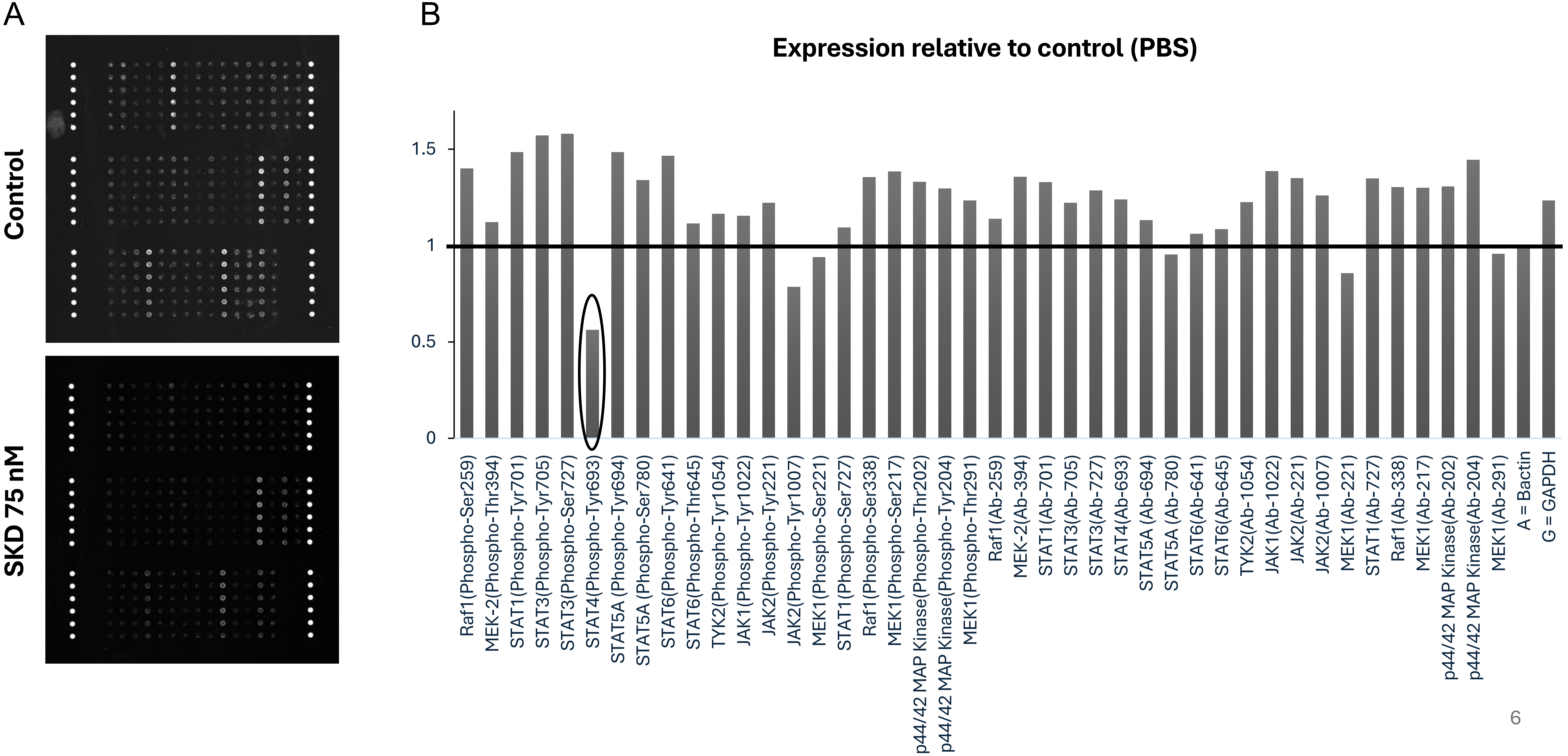

Supplementary Figure S7. Western blot images of the whole membrane

A

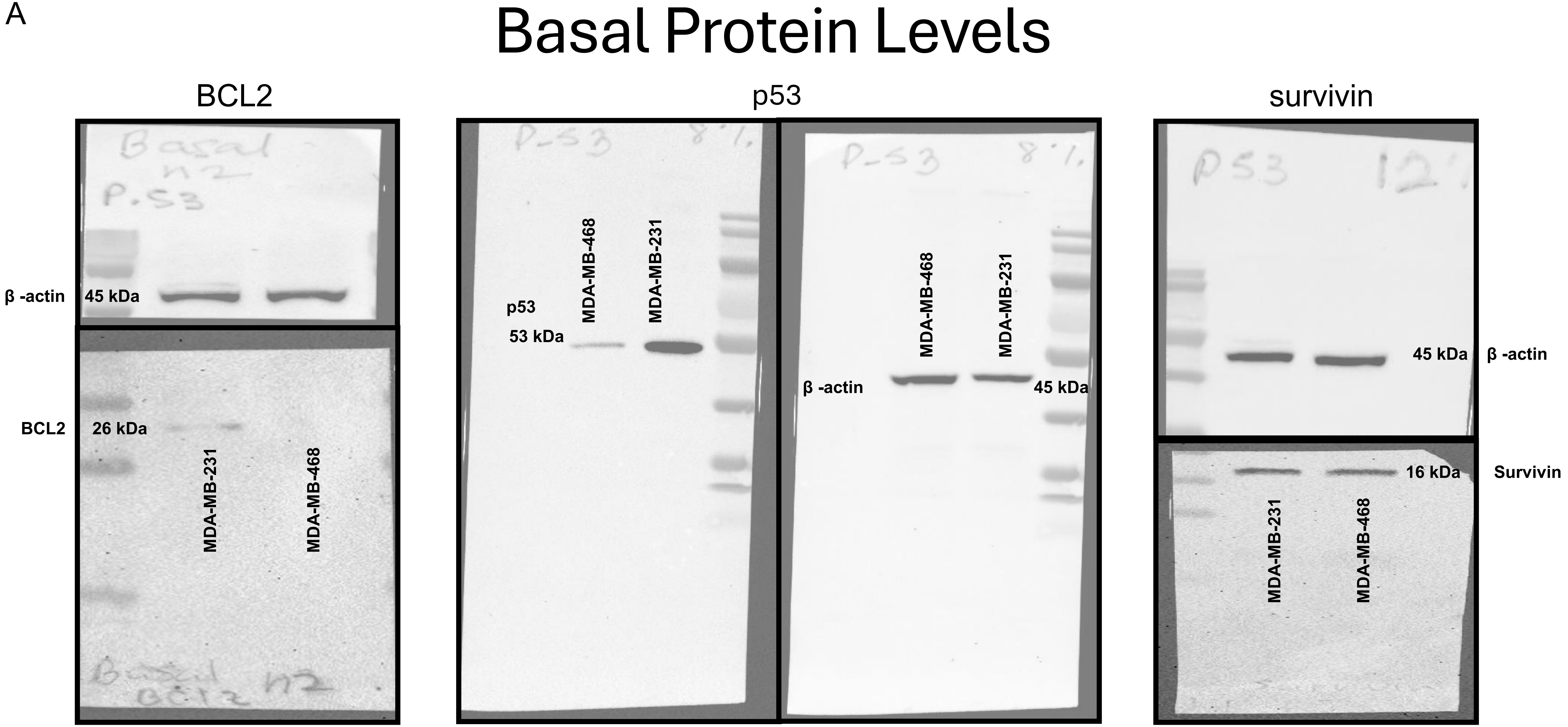

B

Basal Protein Levels

Caspase 3

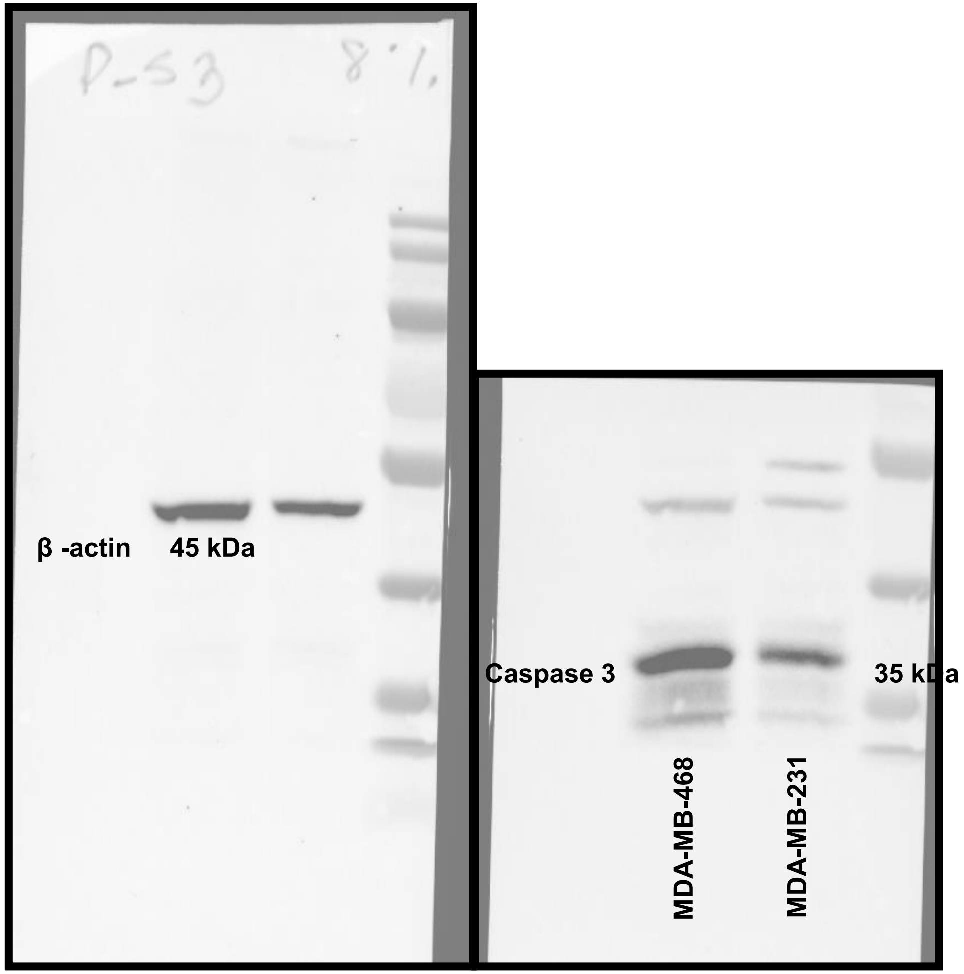

Changes in apoptotic/antiapoptotic protein levels in treated MDA-MB-468 cells

C

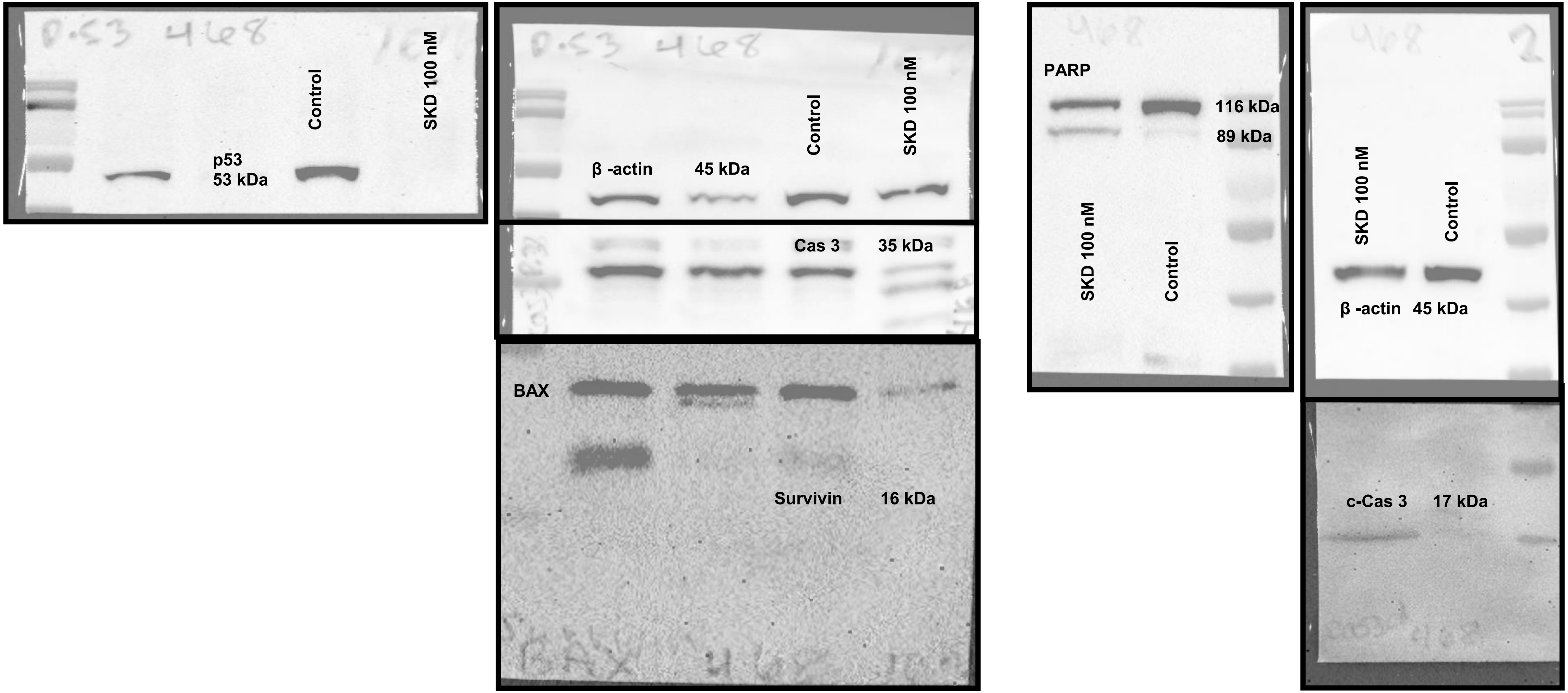

Changes in apoptotic/antiapoptotic protein levels in treated MDA-MB-468 cells

D

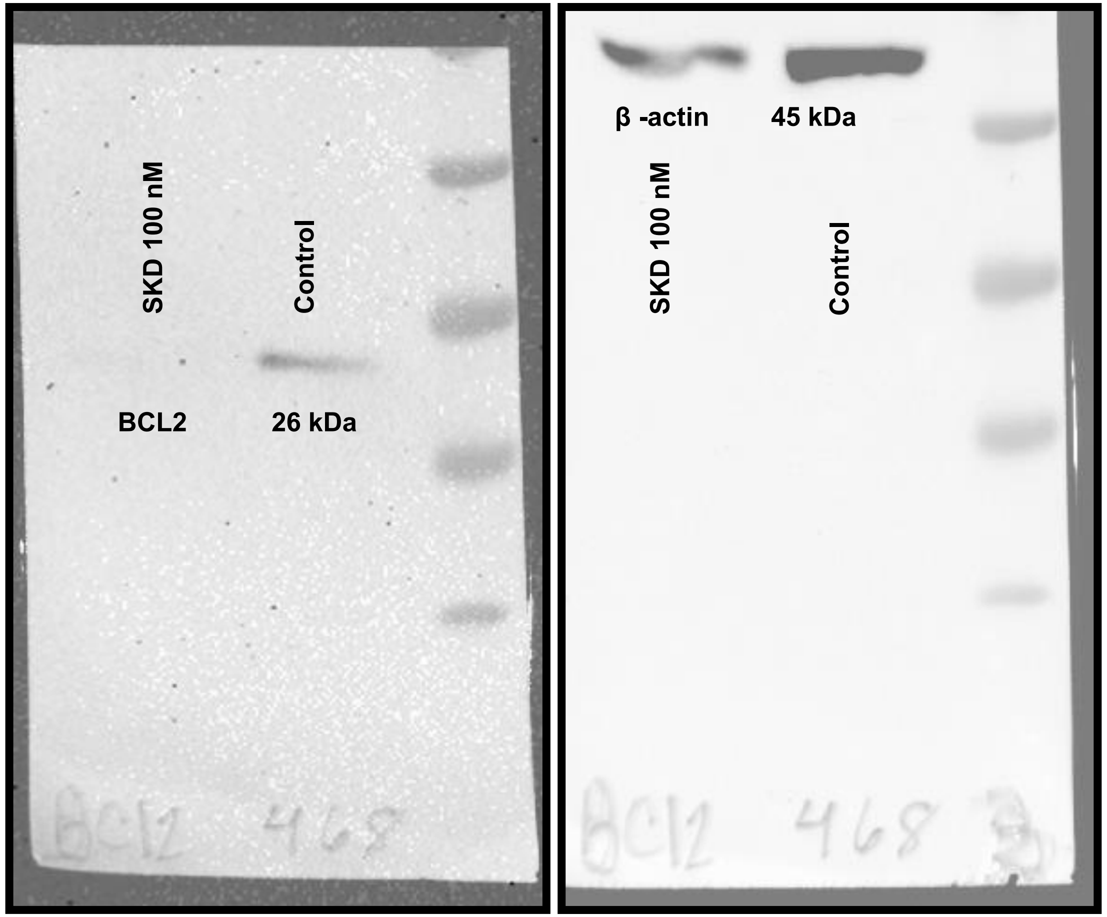

Supplementary Figure S8. Western blot uncropped membranes Corresponding to Figure 6

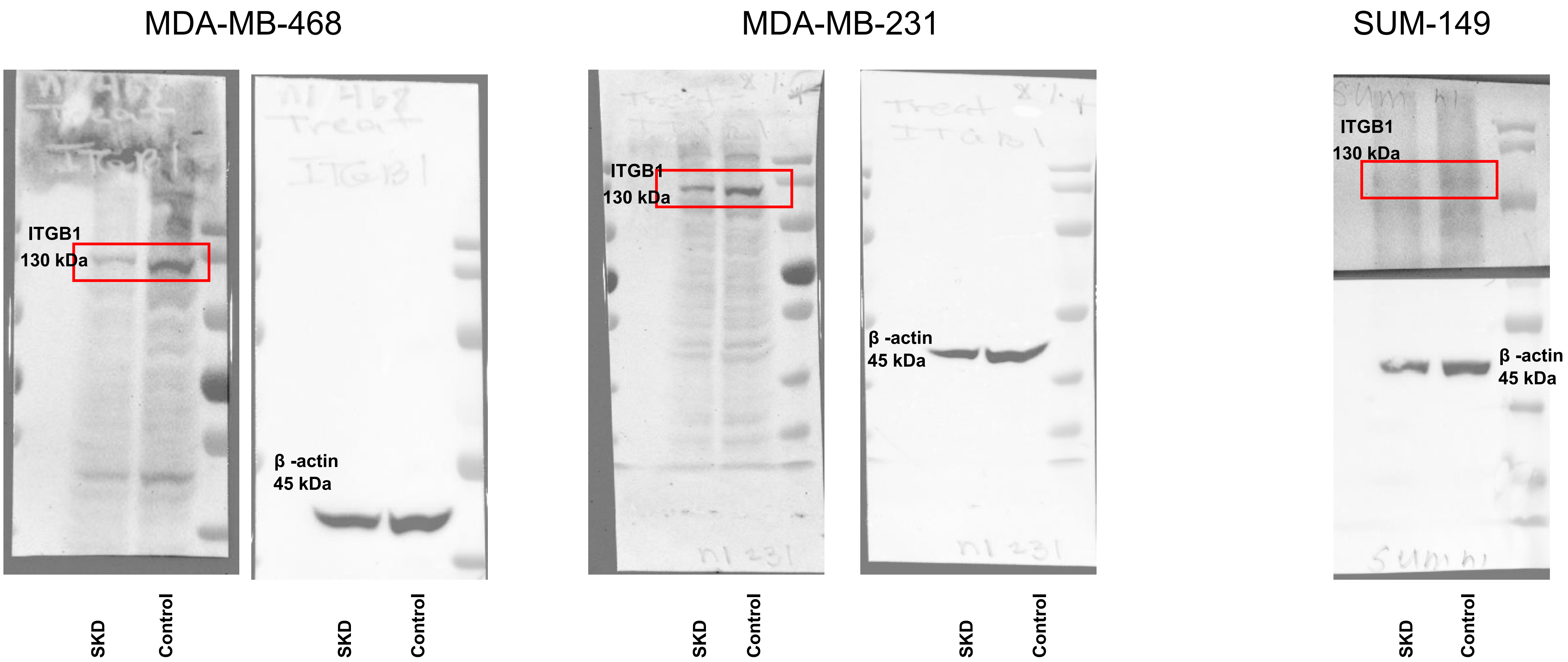

Supplement: Supplementary file 1 [file biomolecules-15-01561-s001.zip › biomolecules-3874416-supplementary.pdf]
